# Constrained graph dynamic spatial perception adversarial network for human motion generation

**DOI:** 10.1371/journal.pone.0339297

**Published:** 2026-01-05

**Authors:** Wanyi Li, Jielin Yang, Jin Li, Yechun Zhao, Yingyin Fan, Yilin Wu, Ling Zou

**Affiliations:** 1 School of Computer Science and Artificial Intelligence, Guangdong University of Education, Guangzhou, P.R. China; 2 School of Management, Guangdong University of Education, Guangzhou, P.R. China; 3 Library, Guangdong University of Education, Guangzhou, P.R. China; 4 College of Information and Intelligence, Hunan Agricultural University, Changsha, P.R. China; Khalifa University of Science and Technology, UNITED ARAB EMIRATES

## Abstract

Accurate 3D skeletal model is fundamental to human pose estimation and body shape reconstruction, as it encodes intricate motion dynamics and spatial configurations. However, generating high-fidelity 3D skeleton samples that adhere to human kinematic constraints remains a significant challenge. To address this problem, the Constrained Dynamic Graph Spatial Perception Adversarial Network (CDGSPAN) is proposed, which is designed to model and synthesize human motion poses with high realism. CDGSPAN leverages dynamic graph-based operations to capture the spatial angular relationships between skeletal joints, while incorporating a constraint-aware regularization mechanism to guide the learning process. This joint modeling enables the network to effectively learn motion priors from real 3D skeletal samples and generate synthetic poses that closely align with biomechanical plausibility. Extensive experiments demonstrate that CDGSPAN achieves superior performance compared to recent adversarial network frameworks in generating sparse 3D skeletal sequences that preserve natural human motion characteristics.

## 1. Introduction

The generation of 3D human motion skeletal model [1] is a prominent research topic in the fields of Artificial Intelligence (AI) and Computer Vision (CV), with significant implications for 3D body shape estimation [2], the development of life like 3D character models [3,4], and virtual reality technologies [5]. However, generating accurate 3D skeletal (keypoint/joint) models of human motion poses is full of numerous technical challenges. These include the high dimensionality of spatial data, the complexity of human skeletal spatial and angular structures, deficiencies in input data, the need to model dynamic and pose variations, and substantial computational demands. Firstly, the high-dimensional nature of 3D human keypoint data requires models to handle complex spatial relationships—capturing not only joint-to-joint spatial dependencies but also the dynamic transitions among different body parts. Furthermore, due to the intricacy of human joints and skeletal anatomy, models must adhere to the physical constraints of joint movement and inter-joint relations to produce valid motion poses. When reconstructing (or estimating) 3D human poses from 2D images [6,7] critical spatial information is lost due to 2D projection, and issues such as noise and occlusion often lead to incomplete input data, compromising accuracy.

Dynamic pose generation further requires models to go beyond static pose prediction and capture temporal-spatial transitions across actions, which involves modeling complex dependencies among keypoints across time. In recent years, Graph Neural Networks (GNNs) [8–10] have gained popularity for processing graph-structured data (composed of nodes and edges), showing potential in modeling the topology and spatial relations of human skeletons. However, conventional graph convolutions in GNNs rely on fixed skeletal connectivity and fail to capture the intrinsic motion patterns of 3D skeleton samples. Even with extensive training, such models often struggle to generate realistic, physically plausible 3D motion sequences. Some GNN variants employ Graph Attention Networks (GATs) [11–13], which have shown promise in various graph-based tasks. Nonetheless, their performance in generating 3D skeletal models remains limited. The primary limitation lies in the coarse modeling of spatial relations—human motion requires fine-grained constraints on joint positions and angular configurations, whereas GATs focus on localized attention computations, which are insufficient to capture the complex spatial dependencies among joints in 3D space.

To address these challenges, the Constrained Dynamic Graph Spatial Perception Adversarial Network (CDGSPAN) is proposed, which offers enhanced flexibility in modeling human motion patterns and the relative spatial configuration of keypoints. CDGSPAN introduces a dynamic graph computation framework for both the generator and discriminator, enabling adversarial learning to progressively optimize the generation of 3D skeletal samples that conform to human motion rules. Unlike existing adversarial networks [14–18], which primarily focus on generating high-dimensional image data (often exceeding hundreds of thousands of pixels), our proposed CDGSPAN is tailored for sparse, structured data that embodies human motion dynamics. CDGSPAN incorporates a spatial perception mechanism capable of capturing the relative joint positions and limb angles of 3D skeletal models through dynamic graph operations. Specifically, edge weights in the graph are updated dynamically based on node features and spatial direction vectors, allowing the model to adaptively handle various poses and joint relationships. This dynamic update mechanism significantly improves the model’s ability to capture meaningful spatial information from each pose sample.

Based on this dynamic computation, the generator and discriminator are constructed to form the adversarial network. During training, we introduce a spatial constraint module that regularizes the loss function, effectively preventing the generation of anatomically implausible limb configurations and improving the realism of synthesized poses. Additionally, we propose an evaluation method to quantitatively assess the validity of generated 3D skeletal samples, independent of visual observation. This method evaluates whether the joint angles and spatial configurations adhere to realistic human kinematics and closely resemble real samples.

In summary, the major contributions of this article are as follows:

1. A new dynamic graph operation is proposed, and a corresponding generator and discriminator are designed to construct the CDGSPAN model.
2. Spatial constraint models are incorporated during the training process, enhancing the model’s understanding of pose geometry and enabling the generation of physically plausible 3D skeletal samples under kinematic constraints.
3. A style-specific sample selection criterion for CDGSPAN that filters generated poses by their Frobenius distance to a reference skeleton under a certain tolerance, and select the representative sample of style-specific sample. This provides a consistent way to obtain style-constrained samples for generation and evaluation.
4. A non-visual evaluation method is developed to assess the quality of generated 3D skeletal data, based on whether the joint angles and spatial positions conform to human motion principles and resemble real-world samples.

The following sections provide a detailed account of the methodology, experiments, and findings.

## 2. Related works

### 2.1. Spatial modeling and spatial constraint models

Many graph-based pose methods still rely on a fixed skeletal topology or coarse, local attention, which limits fine joint-to-joint modeling across different poses. Multi-scale residual GCNs [19] for motion prediction typically operate on predefined skeleton graphs, so edge patterns cannot adapt per pose. Dynamic dense GCNs [20] relax the static-graph assumption, yet they usually do not integrate spatial constraint models into training, leaving geometric validity under-regularized. Evidence that fixed or non-adaptive edges restrict representation also appears outside human pose: a 3D–2D hybrid and GAT model [13] in hyperspectral classification explicitly argues that fixed node weights/edges constrain learning and shows gains from attention-based adaptive edges. Likewise, graph-attention solutions in address matching [11] improve local matching but remain local/coarse and are unrelated to human geometry. Overall, prior work either uses fixed graphs or coarse attention, and seldom employs spatial constraint models to enforce limb relations or angle/length consistency.

### 2.2. Frame-wise spatial representation and sampling stability

Another line of work emphasizes generative quality or distributional fit while omitting explicit constraints on per-frame body geometry. DCNN-based pose generators [21] for animated characters optimize reconstruction/adversarial losses but omit spatial constraint models, which can yield anatomically implausible poses. Flow-based structured prediction [22] improves likelihood and mode coverage yet typically lacks spatial constraint models that suppress per-frame geometric artifact. Diffusion-style generators stress realism/diversity—e.g., a pose-guided diffusion transformer [23] and cross-diffusion motion models [24]—but evaluate mainly with image/video quality or retrieval metrics (FID, R-Precision, etc.), again without explicit spatial constraint models. Several GAN variants [15–17] from adjacent domains (document enhancement, orthogonal subspace disentanglement, differentiable GAN search) likewise provide no built-in safeguards for frame-wise anatomical validity if transferred to skeleton generation, because spatial constraint models are absent by design.

### 2.3. Data robustness and objective (non-visual) evaluation

Most prior evaluations lean on visual or task-level scores and rarely report non-visual criteria that test geometric validity (e.g., joint-angle legality, bone-length consistency, self-intersection checks)—i.e., metrics aligned with spatial constraint models. Image/video pose transfer typically reports LPIPS/SSIM/PSNR or perceptual features rather than limb-level spatial validity [25]. Diffusion models [23,24] report distributional and user-study metrics rather than non-visual spatial tests. Graph-based predictors [19,20] commonly use MPJPE-style geometric errors, which do not certify anatomical plausibility. Broader generative/vision literature—style transfer [26], super-resolution [18], document enhancement [17], text-to-image surveys [14], network embedding [27], radiomics-GANs [28]—centers on visual fidelity, segmentation accuracy, or topology preservation, not non-visual spatial evaluation [11,13,16]. These gaps motivate methods that pair adaptive graph modeling with explicit spatial constraint models and that adopt objective, non-visual metrics to judge whether generated skeletons are spatially valid.

### 2.4. Improving on existing methods

Based on these challenges, we propose the Constrained Dynamic Graph Spatial Perception Adversarial Network (CDGSPAN). This method introduces a dynamic graph computation framework for both the generator and discriminator, enabling adversarial learning to progressively optimize the generation of 3D skeletal samples that adhere to human motion principles. By incorporating spatial constraint models, such as joint-angle limits and bone-length consistency, CDGSPAN addresses the issues found in previous methods and ensures the anatomical plausibility of generated poses. This makes CDGSPAN a significant step forward in addressing the lack of fine-grained motion modeling, the absence of explicit geometric constraints, and the deficiency of objective, non-visual evaluation metrics in existing methods.

## 3. Construction of 3D human motion skeletal model

**Fig 1:**
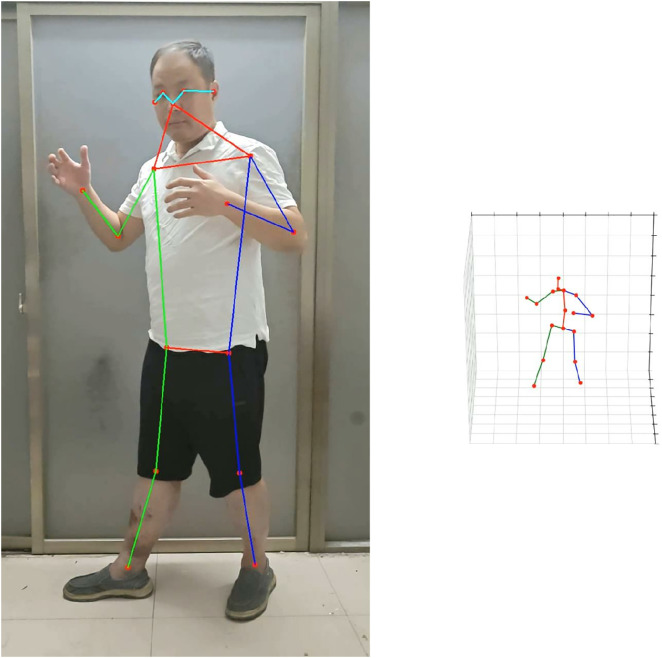
3D skeletal model generated from the captured human motion image keypoints (joints).

**Fig 2:**
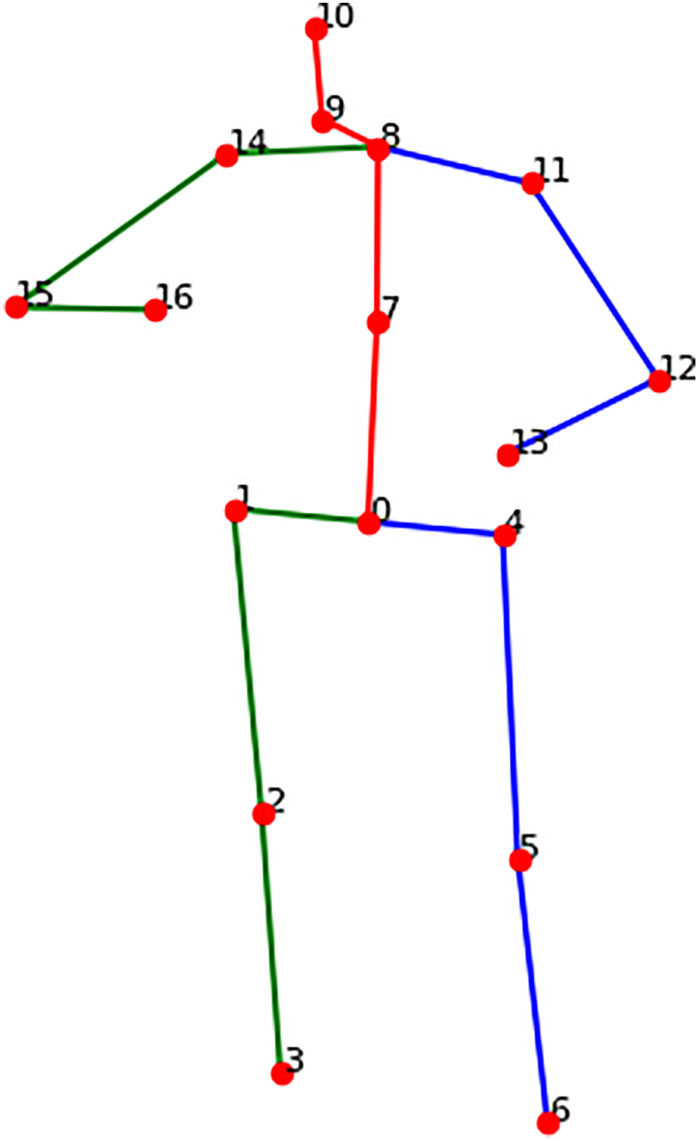
Valid pose model.

A 17 × 3 matrix is used to represent the 3D human skeletal model, where each of the 17 rows corresponds to a uniquely indexed keypoint in 3D space. These keypoints encode the spatial coordinates of human joints and are commonly referred to as keypoints in pose estimation literature. The skeletal data can be obtained either through direct capture from motion sensors or reconstructed from 2D images using deep learning-based pose estimation algorithms [29,30]. As illustrated in Fig 1, connecting these keypoints yields the structural representation of human limbs in the natural daily-life scenarios or videos extracted from standard benchmark datasets such as HumanEva [31] and Human3.6M [32], capturing their relative lengths, spatial positions, and angular orientations. This structure enables intuitive visualization and modeling of diverse human motion patterns, as shown in Fig 2.

**Fig 3:**
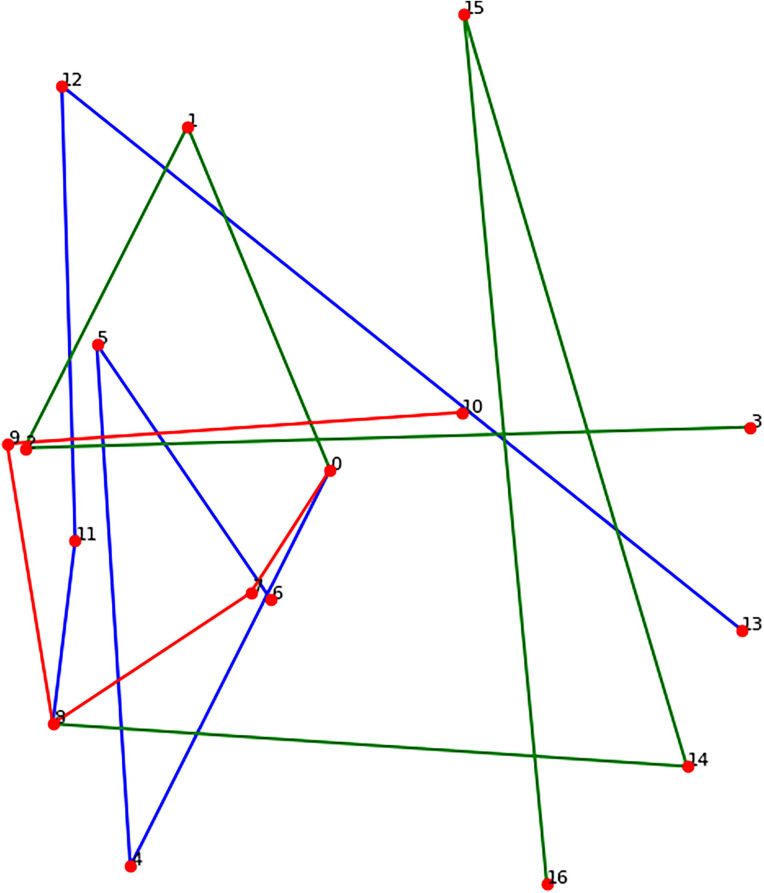
Disordered pose model.

**Fig 4:**
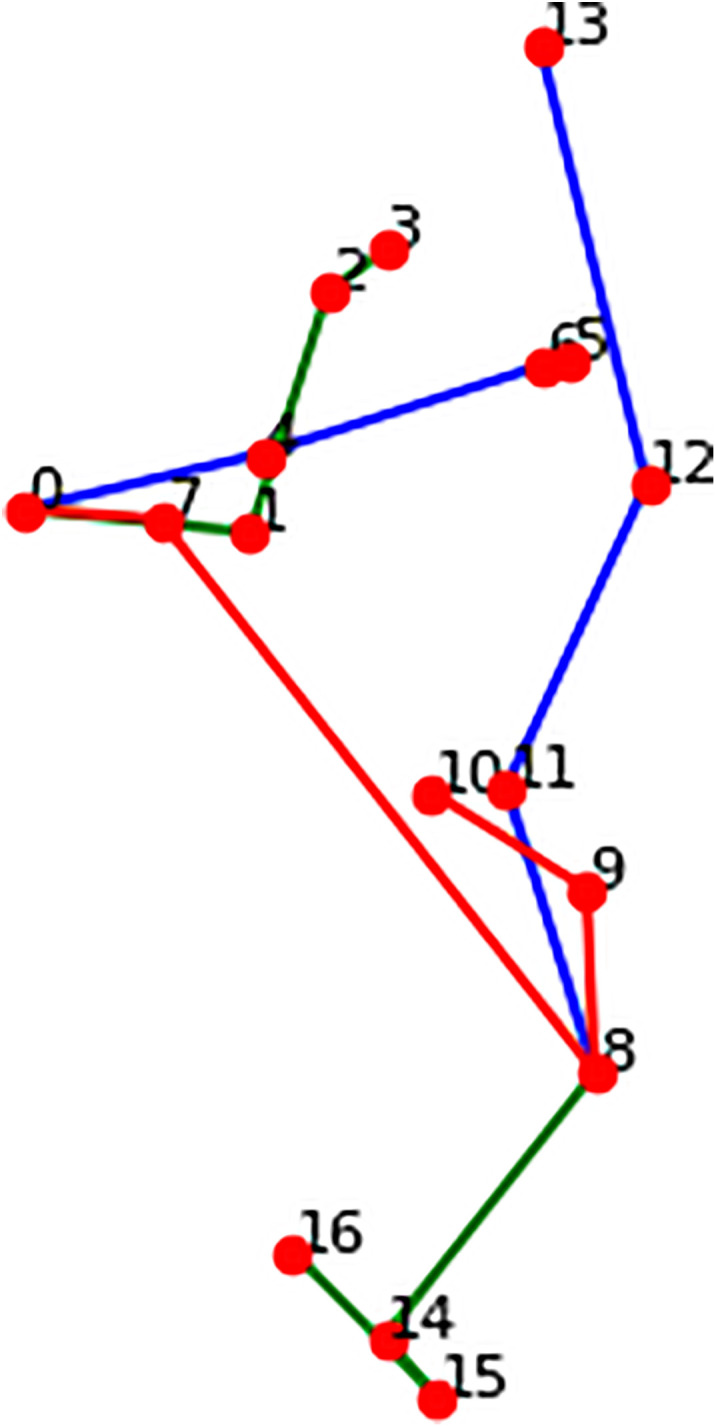
Pose model inconsistent with human kinematic rules.

The spatial positioning of each keypoint is critical, as it encodes the semantic logic of human pose. Different joint angles and spatial configurations reflect different motion states. However, if the keypoints are arranged in a disordered or anatomically implausible manner—for example, when the hands, feet, or head are misaligned or reversed—the resulting 3D pose is invalid, as illustrated in Figs 3 and 4 Therefore, accurate modeling of the relative spatial relationships among keypoints is essential for training models that can generate valid and realistic 3D human poses.

**Fig 5:**
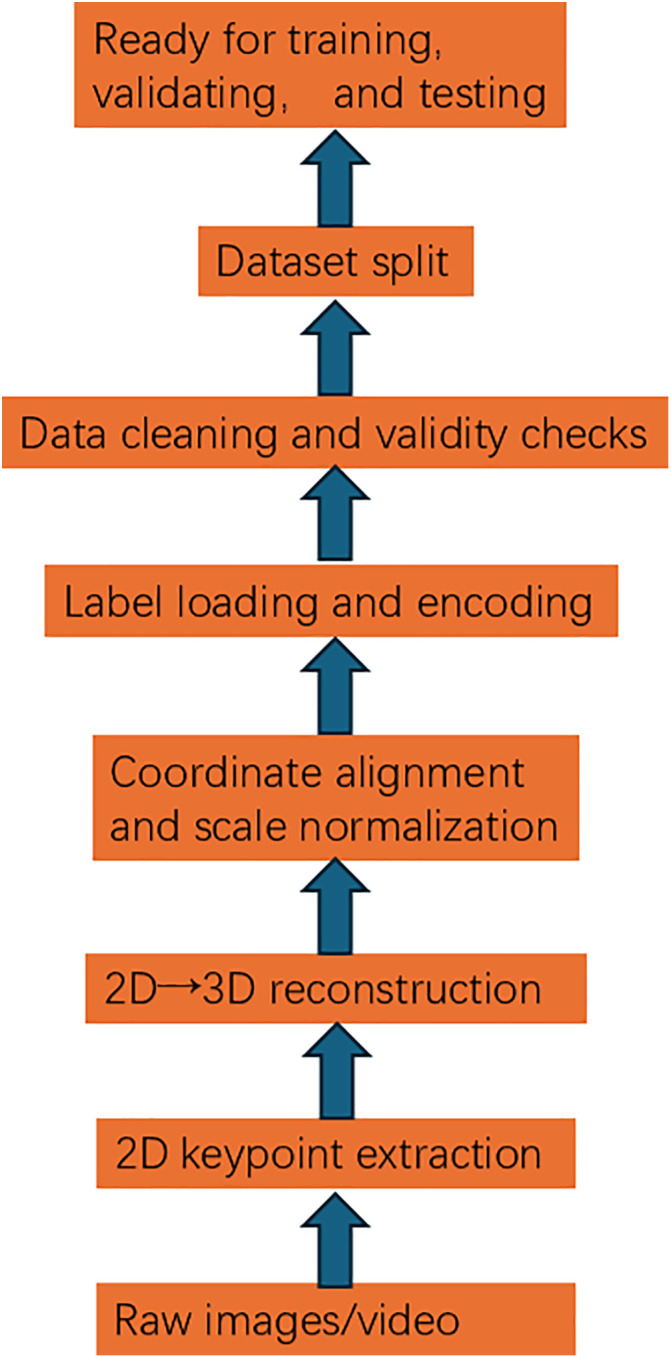
Preprocessing workflow.

An end-to-end preprocessing workflow is adopted to convert raw acquisitions into analyzable 3D skeleton sequences, as shown in Fig 5. Starting from raw images/videos, 2D joints are first extracted per frame by a standard pose detector, and basic 2D quality control is applied (filtering low-confidence joints and optional temporal smoothing). Depending on data conditions, 3D poses are then obtained: with multi-view data, camera calibration and triangulation are performed; with monocular data, a 2D-to-3D lifting network is used. The resulting 3D coordinates are centered on a reference joint and scale-normalized to remove body-size effects and maintain consistent bone lengths. Metadata are loaded and class labels (e.g., action/style) are encoded as integer IDs. Data cleaning and validity checks are conducted to remove NaN/Inf entries and poses that clearly violate anatomical constraints (e.g., extreme limb lengths), while class balance is preserved as much as possible. Finally, the dataset is split—preferably stratified and, when necessary, grouped by subject/scene—into training/validation/test sets to ensure reproducibility and prevent information leakage. Through this workflow, scale-consistent and anatomically plausible 3D skeleton data, along with fair and comparable splits, are prepared for model training and evaluation.

## 4. CDGSPAN model

### 4.1. Model architecture and computational principles

**Fig 6:**
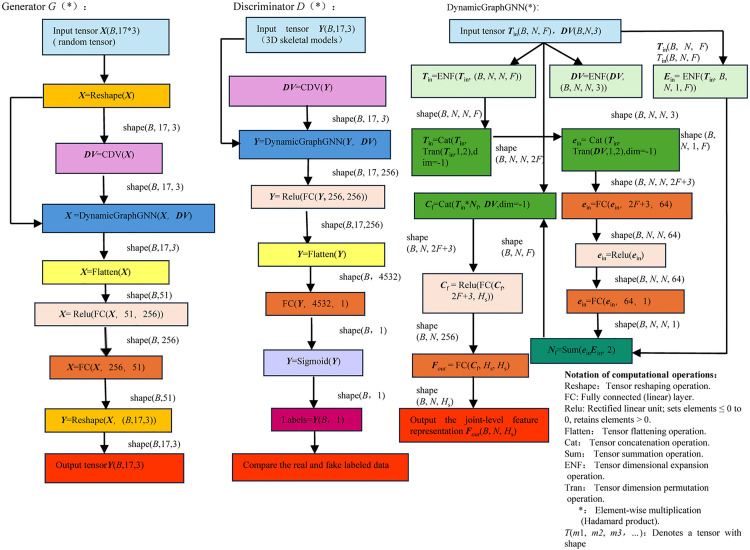
Main computational architecture of CDGSPAN model.


The overall architecture of the proposed CDGSPAN model is illustrated in Fig 6. Some of the key computational definitions are presented as follows. The CDV function is used to calculate the relative position encoding of 3D skeletal model keypoints with respect to a designated root joint. It is defined as:

$$DV=\text{CDV(}X\text{)}\text{=}X-x_{\text{ref}}⊗1_{N},\text{\textbackslash{}hspace\{0.17em}\;\}X\in {\text{R}}^{B\times N\times F},\text{\textbackslash{}hspace\{0.17em}\;\}x_{\text{ref}}=X[:,0,:in{\text{R}}^{B\times F},\text{\textbackslash{}hspace\{0.17em}\;\}1_{N}\in {\text{R}}^{N\times 1}$$ (1)

where $DV\in {\text{R}}^{B\times N\times F}$, represents the relative position (direction vector) of each keypoint with respect to the root joint, and $x_{\text{ref}}$ denotes the reference (root) joint features extracted from the input ***X***. ⊗ denotes tensor broadcasting. The reference joint vector $x_{\text{ref}}$ is replicated *N* times along the joint dimension, resulting in $x_{\text{ref}}⊗1_{N}\in {\text{R}}^{B\times N\times F}$. Next, the DynamicGraphGNN function *F*_out_=DynamicGraphGNN (*) performs the proposed dynamic graph operation to estimate spatially adaptive relations among skeletal keypoints. The relative spatial distances between nodes are encoded as follows. First, we compute pairwise relative features between all joints, then perform a one-dimensional concatenation across the feature vectors:


E=Cat(X,Tran(X,1,2),Dv,dim=−1) (2)

This results in edge feature representations $E\in {\text{R}}^{B\times N\times N\times (2F+3)}$. After that, a multi-layer perceptron (MLP) is applied to compute the dynamic edge weights:


$$f_{\text{edge}}\left(E\right)=\sigma (EW_{\text{1}}+b_{\text{1}})W_{\text{2}}+b_{\text{2}}$$ (3)

where $W_{\text{1}}\in {\text{R}}^{(2F+3timesH},\text{\textbackslash{}hspace\{0.17em}\;\}b_{\text{1}}\in {\text{R}}^{B\times N\times N\times H},\text{\textbackslash{}hspace\{0.17em}\;\}W_{\text{2}}\in {\text{R}}^{H\times 1},\text{\textbackslash{}hspace\{0.17em}\;\}b_{\text{1}}\in {\text{R}}^{B\times N\times N\times 1}$, and σ(·) denotes the ReLU activation function. From the equation (3), The computed edge weights $f_{\text{edge}}\left(E\right)\in {\text{R}}^{B\times N\times N}$ are then expanded into a 4D tensor ${f^{\prime }}_{\text{edge}}\left(E\right)=\text{ENF(}f_{\text{edge}}\left(E\right),\left(B,N,N,F\right)\text{)}\in {\text{R}}^{B\times N\times N\times F}$ and used to modulate spatial information passing through the dynamic graph. The original input tensor is also processed with the similar expansion in the third dimension, resulting in $X^{\prime }=\text{ENF(}X,\left(B,N,N,F\right)\text{)}\in {\text{R}}^{B\times N\times N\times F}$.These representations are used in subsequent modules to compute the weighted neighbor features for each joint:


$$N_{\text{f}}=\sum_{i,j,k=1,l}^{N}({f^{\prime }}_{\text{edge}}(E)⊙X^{\prime })[i,j,k,l]$$ (4)

from Equation (4), the tensor $N_{\text{f}}\in {\text{R}}^{B\times N\times N}$ is obtained to represent the weighted neighbor features for each joint. To integrate multi-source spatial information, the tensors $N_{\text{f}}\in {\text{R}}^{B\times N\times N}$, the original input ***X***, and the relative position tensor ***DV*** are concatenated along the last dimension. As a result, a fused feature tensor is constructed, as defined in Equation (5):


$$C_{\text{f}}=\text{Cat}(N_{\text{f}},X,DV,dim=-1)$$ (5)

In Equation (5), Cat is denoting the tensor concatenation operation along dimension (dim); we set dim = −1 to indicate the last dimension. The tensor $C_{\text{f}}\in {\text{R}}^{B\times N\times \text{(}2F+3)}$ is subsequently passed through a two-layer fully connected network to compute the final joint-level feature representation, as formulated in Equation (6):


$$F_{\text{out}}=\sigma (C_{\text{f}}W_{\text{3}}+b_{\text{3}})W_{\text{4}}+b_{\text{4}}$$ (6)

where, $W_{\text{3}}\in {\text{R}}^{(2F+3timesH}$ and $W_{\text{4}}\in {\text{R}}^{H\times H}$ denote the learnable weight matrices, $b_{\text{3}},b_{\text{4}}\in {\text{R}}^{B\times N\times H}$ are the corresponding bias terms. The ReLU activation function σ(·) is employed between the two layers. Consequently, the output feature tensor $F_{\text{out}}\in {\text{R}}^{B\times N\times H}$ is obtained, which encodes the spatial representations of each joint by incorporating both individual joint features and context from neighboring keypoints.

**Fig 7:**
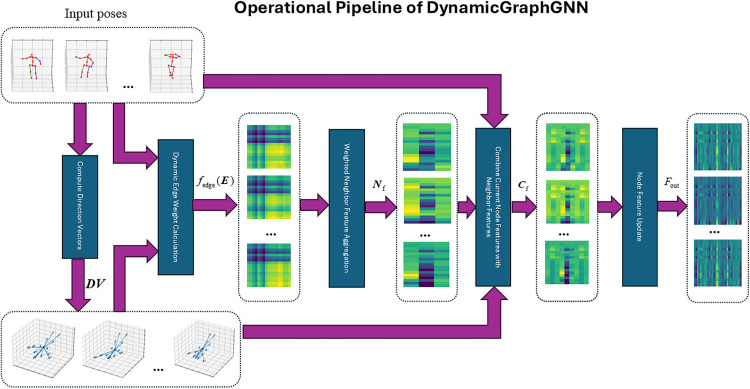
Operational pipeline of DynamicGraphGNN (Pose samples are used as input).

The operational pipeline of DynamicGraphGNN can be seen in Fig 7. Given an input sequence of poses, we first compute direction vectors (***DV***) between relevant joints to encode local geometry. These vectors parameterize a dynamic edge-weight function $f_{\text{edge}}\left(E\right)$, producing stochastically varying weight matrices that adapt the graph topology per frame. Using these weights, the network performs weighted neighbor feature aggregation to obtain $N_{\text{f}}$, which is then combined with each node’s current features to form $C_{\text{f}}$. A node feature update module (e.g., linear/MLP with residual gating) outputs the refined representations ***F***_out_. The purple arrows indicate that this process is applied recurrently across frames, enabling adaptive and geometry-aware message passing.

The DynamicGraphGNN accepts either a pose sequence $Y\in {\text{R}}^{B\times N\times F}$ together with the root-relative direction vectors (*DV*)computed with respect to the first (root) joint, or a random tensor $X\in {\text{R}}^{B\times N\times F}$ with the same total number of elements as the pose sequence (possibly a different shape), paired with the same root-relative direction vectors. In both cases, the direction vectors are obtained by subtracting the coordinates of the root joint from those of all joints in each frame (i.e., vectors pointing from the root to each joint). The representations produced by DynamicGraphGNN are fed into task-specific linear heads. In th**e** generator *G*(*), a linear projection maps the features to a new pose sample. In the discriminator *D*(*), a linear classifier outputs a real/fake label indicating whether the generated pose sample is consistent with the training samples.

### 4.2. Construction of the spatial constraint models

In adversarial learning, constraints are built to ensure that the generated 3D skeletal pose samples not only satisfy the discriminator’s classification criteria, but also conform to the physical and biomechanical principles of real human motion. In particular, for pose generation tasks, it is essential that the positions and angles of the generated joints adhere to human kinematic constraints, thereby avoiding the generation of unnatural or physically implausible poses.

By incorporating **s**patial position constraints, the generator is guided to maintain reasonable distances between adjacent joints, which helps prevent the generation of joint pairs that are either excessively close or unnaturally far apart. In parallel, angular constraints are applied to ensure that the angle variations between connected joints remain within physiologically feasible ranges. For example, the bending angles of the elbow and knee joints must not exceed human anatomical limits. These constraints not only improve the plausibility and realism of the generated poses, but also help stabilize the generator and mitigate issues such as mode collapse during training. By integrating spatial and angular constraints into the adversarial learning framework, the generator is enabled to produce physically valid and kinematically consistent poses while maintaining high visual fidelity. This integration enhances training efficiency and model usability. Moreover, the inclusion of such constraints improves model stability, reduces convergence time, and ensures that the generated poses are not only visually realistic but also practically viable for downstream applications.

To enforce these constraints, regularization terms are incorporated into the generator’s loss function during training. These regularization terms act as guiding signals, steering the generator to comply with predefined spatial and kinematic rules and thus improving the quality of the generated 3D skeletal samples.

In the following, we define two essential constraint formulations for pose modeling.

The 3D skeletal model used in this work (as shown in Fig 2) is subject to inter-joint distance limitations. Since the skeleton has a predefined joint connection structure, a set of connected joint pairs can be defined as:


$$\begin{array}{c} C_{\text{kp}}=\{p_{\text{0}}=(0,4),p_{\text{1}}=(4,5),p_{\text{2}}=(5,6),\#\text{left leg}\\ p_{\text{3}}=\left(8,11\right),p_{\text{4}}=\left(11,12\right),p_{\text{5}}=\left(12,13\right),\#\text{right arm}\\ p_{\text{6}}=\left(0,1\right),p_{\text{7}}=\left(1,2\right),p_{\text{8}}=\left(2,3\right),\#\text{right leg}\\ p_{\text{9}}=\left(8,14\right),p_{10}=\left(14,15\right),p_{11}=\left(15,16\right),\#\text{left arm}\\ p_{12}=\left(0,7\right),p_{13}=\left(7,8\right),p_{14}=\left(8,9\right),p_{15}=\left(9,10\right),\#\text{torso to head}\} \end{array}$$ (7)

From Equation (7), the set $C_{\text{kp}}$ defines 16 connections between the 17 joints in the 3D skeletal model, where each *p*_*i*_, represents the distance between a pair of connected joints. Therefore, let $K_{\text{G}}\in {\text{R}}^{B\times 17\times 3},\text{\textbackslash{}hspace\{0.33em}\}K\in {\text{R}}^{B\times 17\times 3}$ denote the generated and real 3D skeletal samples for a batch *B* of training data respectively. A spatial distance constraint model can be established as follows:


$$L_{\text{spatial}}=\frac{\text{1}}{17B}\sum_{b=1}^{B}\sum_{i=0}^{15}\left|\left|\right|K_{\text{G}}\text{(}b\text{,}p_{i}\text{(0),:)-}K_{\text{G}}\text{(}b\text{,}p_{i}\text{(1),:)||}-\left|\right|K\text{(}b\text{,}p_{i}\text{(0),:)-}K\text{(}b\text{,}p_{i}\text{(1),:)||}\right|=0$$ (8)

In Equation (8), let the i-th skeletal edge be $p_{i}=\left(p_{i}(\text{0}),p_{i}(\text{1})\right)$. Define the limb vectors $\Delta K_{\text{G}}\left(b,p_{i}\right)=K_{\text{G}}\left(b,p_{i}\left(0\right),:\right)-K_{\text{G}}\left(b,p_{i}\left(1\right),:\right),\text{\textbackslash{}hspace\{0.17em}\;\}\Delta K\left(b,p_{i}\right)=K\left(b,p_{i}\left(0\right),:\right)-K\left(b,p_{i}\left(1\right),:\right).$ Equation (8) is expressed as the following equality constraints (bone-length consistency): $g_{b,i}\left(K_{\text{G}}\right)=|∥\Delta K_{\text{G}}\left(b,p_{i}\right)∥-∥\Delta K\left(b,p_{i}\right)∥|=\text{0},\forall b,\;i=\text{0},\ldots ,\text{15}$. Using joint triplets, a set of angular constraint joint groups is defined as:


$$\begin{array}{c} \begin{array}{c} A_{\text{c}}\text{\textbackslash{} =\textbackslash{} }\{a_{\text{0}}\text{\textbackslash{} =\textbackslash{} (0, 4, 5),}a_{\text{1}}\text{\textbackslash{} =\textbackslash{} (4, 5, 6),}a_{\text{2}}\text{\textbackslash{} =\textbackslash{} (8, 11, 12),}a_{\text{3}}\text{\textbackslash{} =\textbackslash{} (11, 12, 13),}\\ a_{\text{4}}\text{\textbackslash{} =\textbackslash{} (0, 1, 2),}a_{\text{5}}\text{\textbackslash{} =\textbackslash{} (1, 2, 3),}a_{\text{6}}\text{\textbackslash{} =\textbackslash{} (8, 14, 15),}\\ a_{\text{7}}\text{\textbackslash{} =\textbackslash{} (14, 15, 16),}a_{\text{8}}\text{\textbackslash{} =\textbackslash{} (0, 7, 8),}a_{\text{9}}\text{\textbackslash{} =\textbackslash{} (7, 8, 9),}a_{10}\text{\textbackslash{} =\textbackslash{} (8, 9, 10)}\} \end{array} \end{array}$$ (9)

Then, the cosine similarity between the generated and ground-truth limb directions is computed using Equation (10) and Equation (11):


$$cos\theta_{\text{G}}^{b}=\sum_{i=0}^{10}\frac{(K_{\text{G}}\text{(}b\text{,}a_{i}\text{(0),:)-}K_{\text{G}}\text{(}b\text{,}a_{i}{\text{(1),:))}}^{\text{T}}((K_{\text{G}}\text{(}b\text{,}a_{i}\text{(2),:)-}K_{\text{G}}\text{(}b\text{,}a_{i}\text{(1),:))}}{\left|\right|\left(K_{\text{G}}\text{(}b\text{,}a_{i}\text{(0),:)-}K_{\text{G}}\text{(}b\text{,}a_{i}\text{(1),:))||}\right||(K_{\text{G}}\text{(}b\text{,}a_{i}\text{(2),:)-}K_{\text{G}}\text{(}b\text{,}a_{i}\text{(1),:)||}}$$ (10)

$$\begin{array}{c} cos\theta_{\text{R}}^{b}=\sum_{i=0}^{10}\frac{(K\text{(}b\text{,}a_{i}\text{(0),:)-}K\text{(}b\text{,}a_{i}{\text{(1),:))}}^{\text{T}}((K\text{(}b\text{,}a_{i}\text{(2),:)-}K\text{(}b\text{,}a_{i}\text{(1),:))}}{\left|\right|\left(K\text{(}b\text{,}a_{i}\text{(0),:)-}K\text{(}b\text{,}a_{i}\text{(1),:))||}\right||(K\text{(}b\text{,}a_{i}\text{(2),:)-}K\text{(}b\text{,}a_{i}\text{(1),:)||}} \end{array}$$ (11)

Subsequently, the angular error between the generated data and the ground-truth data is computed as:


$$L_{\text{angle}}=\sum_{b=1}^{B}max(0,(\left|cos\theta_{\text{G}}^{b}-cos\theta_{\text{R}}^{b}\right|-\epsilon )=0,\epsilon \in [0,0\mathrm{.01}]$$ (12)

In Equation (12), *ε* denotes the acceptable angular deviation margin, this constraint is equivalent to $\begin{array}{c} cos\theta_{G}^{b}-cos\theta_{R}^{b}|\leq \epsilon ,\text{\textbackslash{}hspace\{0.17em}\;\}\forall b,\text{\textbackslash{}hspace\{0.17em}\;\}\epsilon \in [0,0\mathrm{.01}] \end{array}$. If the deviation is smaller than *ε*, no loss is applied. These constraints define the manifold $ℳ=\left\{K_{\text{G}}\text{\textbackslash{}hspace\{0.33em}|\;\}g_{b,i}\left(K_{\text{G}}\right)=0,\text{\textbackslash{}hspace\{0.33em}\}\left|\text{cos}\theta_{\text{G}}^{b}-\text{cos}\theta_{\text{R}}^{b}\right|\leq \epsilon \right\}$.

### 4.3. Construction of the training procedure

Based on Sections 4.1 and 4.2, the training procedure can be constructed as illustrated in Fig 6. First, the loss functions for the generator and discriminator are defined as follows:


$$K_{\text{G}}=G\left(Z\right)\in {\text{R}}^{B\times 17\times 3},Z\in {\text{R}}^{B\times 17\times 3},Z\left(b,i,j\right)\;N\left(0,1\right)$$ (13)

$$L_{f_{\text{D}}}=-E_{K}[{log}_{e}D(K)]-E_{K_{\text{G}}}[{log}_{e}(1-D(G(Z)))],K\in {\text{R}}^{\text{B}\times 17\times 3}$$ (14)

$$L_{f_{\text{G}}}=-E_{Z}[{log}_{e}D(G\left(Z\right))]$$ (15)

The full objective of the generator and discriminator is then defined as:


$${L^{\prime }}_{f_{\text{G}}}=L_{f_{\text{G}}}+\lambda_{\text{spatial}}L_{\text{spatial}}+\lambda_{\text{angle}}L_{\text{angle}}$$ (16)

Equations (13) through (16) are established to guide the optimization of model parameters *f*_D_, *f*_G_ during training. The training process is based on binary cross-entropy (BCE) loss, with the final generator objective defined in Equation (16), which incorporates regularization terms on spatial and angular constraints, weighted by the hyperparameters *λ*s_patial_, *λ*_angle_ respectively. During training, the discriminator D(K) is optimized to maximize the probability of correctly identifying real data, i.e., assigning a confidence score close to 1 for real samples. Simultaneously, the discriminator D(G(Z\leftright) is trained to minimize the likelihood of mistakenly classifying generated data as real, pushing the predicted probability for fake data (i.e., samples generated by the generator) toward 0. Then, the generator G(Z) is trained to minimize the overall loss ${L^{\prime }}_{f_{\text{G}}}$, such that the generated 3D skeletal samples not only adhere to human joint spatial and kinematic constraints but also successfully deceive the discriminator, thereby making the output probability from D(G(Z\leftright) as close to 1 as possible. Ultimately, the training process aims to produce generated skeletal data that is structurally and spatially close to real human motion samples. Through the adversarial process, the model parameters *f*_D_ and *f*_G_ are iteratively optimized. The pseudo-code of the complete training procedure is provided in Table 1.

**Table 1:** Training pseudo-code of the CDGSPAN model.

Definitions of training parameters: ($randn\left(m_{\text{1}},m_{\text{2}},m_{\text{3}}\right)$ randn(m1, m2, m3) denotes a tensor operation that generates a random tensor of shape $m_{\text{1}}\times m_{\text{2}}\times m_{\text{3}}$, $\nabla_{f}\left(L_{f}\right)$ represents the gradient of the loss function $L_{f}$ used for backpropagation.)	
**Initialization:** initialize the generator G and discriminator D models set the optimizer parameters batch size = 512 learning rate = 0.00001 total number of training epochs: Tepoch = 500 **Training:** For epoch in range(1, Tepoch): Z=randn(B,17,3) $X_{\text{fake}}=G\left(Z\right)$ $L_{f_{\text{D}}}=-E_{X_{\text{real}}}[{log}_{e}D(X_{real}\mathrm{\backslash leftright}]-E_{X_{\text{fake}}}[{log}_{e}(1-D(G(Z)))]$ $f_{\text{D}}=f_{\text{D}}-\eta_{f_{\text{D}}}\nabla_{f_{\text{D}}}\left(L_{f_{\text{D}}}\right)$	$L_{f_{\text{G}}}=-E_{Z}[{log}_{e}D(G\left(Z\mathrm{\backslash rightleft})\right]$ $f_{\text{G}}=f_{\text{G}}-\eta_{f_{\text{G}}}\nabla_{f_{\text{D}}}\left(L_{f_{\text{G}}}\right)$ If epoch % 5== 0: save fD, fG; If generated pose is visually close to the ground-truth pose: Break; End for

Equation (16) can be connected to Lagrange-multiplier dynamics. The holonomic (bone-length) residual is ${\;g}_{b,i}\left(K_{\text{G}}\right)=|∥\Delta K_{\text{G}}\left(b,p_{i}\right)∥-∥\Delta K\left(b,p_{i}\right)∥|=\text{0}$, and the two inequality residuals are $h_{b}\left(K_{\text{G}}\right)=\pm \left(\text{cos}\theta_{\text{G}}^{b}-\text{cos}\theta_{\text{R}}^{b}\right)-\epsilon$ from Equation (12). We set $L_{\text{spatial}}=\sum_{b,i}g_{b,i}{\left(K_{G}\right)}^{\text{2}},\text{\textbackslash{}hspace\{0.17em}\}L_{\text{angle}}=\sum_{b}{\left(h_{b}{\left(K_{G}\right)}_{+}\right)}^{\text{2}},v_{+}=max\left(0,v\right).\text{\textbackslash{} }$ Jacobians. For a vectorized pose $q=\text{vec}\left(K_{G}\right)\in R^{\text{3}J}$, $J_{g}\left(q\right)=\frac{\partial g\left(q\right)}{\partial q},\text{\textbackslash{}and\textbackslash{}hspace\{0.17em}\}J_{h}\left(q\right)=\frac{\partial h\left(q\right)}{\partial q}$ are the Jacobians of the equality and inequality residual vectors, *J* is the number of joints. Gradient step (discrete constrained dynamics) is $\nabla L_{f_{G}}\text{'}=\nabla L_{f_{G}}+2\lambda_{\text{spatial}}J_{g}^{⊤}g+2\lambda_{\text{angle}}J_{h}^{⊤}\text{h},$ which matches discrete dynamics under Lagrange forces by identifying $\lambda =2\lambda_{\text{spatial}}g,\mu =2\lambda_{\text{angle}}h\;\left(\mu \geq 0\right).$ In an augmented-Lagrangian variant, updating the duals by $\mu \leftarrow {\left[\mu +\rho h\left(K_{\text{G}}\right)\right]}_{+},\lambda \leftarrow \lambda +\rho g\left(K_{\text{G}}\right)$, yields the standard primal–dual scheme. At inference (optional projection), a one-step equality projection can be applied to sharpen feasibility $K_{\text{G}}\leftarrow \hat{K_{\text{G}}}-J_{g}^{⊤}{\left(J_{g}J_{g}^{⊤}+\rho I\right)}^{-1}g\left(\hat{K_{\text{G}}}\right),\rho \in \left[10^{-3}{,10}^{-1}\right]$ with active inequalities incorporated if needed.

In this work, a subset of video sequences from the Human3.6M dataset [32] is selected to construct the training set. For each video, 2D keypoints are extracted using a keypoint detection model [29,30] like the work of Fig 1, and the corresponding 3D skeleton sequences are reconstructed through a pretrained lifting network (e.g., VideoPose3D).

The reconstructed 3D samples are used to train the proposed model. A total of ten human motions are included, such as directions, greeting, phoning, waiting, sitting and so on. 80% of the reconstructed 3D skeletons are selected randomly to train the proposed model, while the remaining 20% is reserved for validation and testing. This split ensures that the model is exposed to diverse motion sequences during training and ensures that its generalization ability is evaluated on unseen samples. The frame statistics for each pose category are summarized in Table 2. A total of 16180 frames are reconstructed and used for training and evaluation. The runtime environment for training, validation, and testing is shown in Table 3

**Table 2:** Dataset (Human3.6M) for the training.

Pose Category	Video File Name	Frame Count
Directions	Directions 1.55011271.mp4	1560
Greeting	Greeting.55011271.mp4	1812
Phoning	Phoning 1.60457274.mp4	3822
Photo	Photo 1.60457274.mp4	1878
Purchases	Purchases.60457274.mp4	1042
Sitting	Sitting.55011271.mp4	2182
Smoking	Smoking.55011271.mp4	2414
Waiting	Waiting 1.55011271.mp4	2283
WalkDog	WalkDog.58860488.mp4	1438
WalkTogether	WalkTogether 1.60457274.mp4	1794
Total		20225
Trianing Frames (80% of Total)		16180

**Table 3:** Training and testing environment parameters.

Item	Configuration
**Operating System**	Windows 11 (Home Chinese Edition)
**Processor**	Intel(R) Core(TM) Ultra 9 275HX @ 2.70GHz
**Memory**	32 GB RAM
**GPU Model & Capacity**	NVIDIA GeForce RTX 5080 Laptop GPU, 16GB
**Deep Learning Toolbox**	PyTorch 2.7.1, CUDA 12.8
**Python**	Python 3.11.13
**Storage Capacity**	4TB SSD
**Training Epochs**	1000 ~ 2000
**Batch Size**	256 ~ 2048
**Learning Rate**	1e-6 ~ 1e-4
**Optimizer**	Adam
**Loss Function**	BCELoss

Our proposed generator and discriminator operate on sparse 3D skeletons (17 × 3 matrices) rather than high-dimensional RGB images. Feasible human poses lie on a kinematics-constrained low-dimensional manifold, which reduces sample complexity. Beyond data size, CDGSPAN uses explicit skeletal topology and spatial/angle constraints as structural regularizers. Early stopping and learning-rate scheduling are employed to reduce the risk of overfitting, and training stability is maintained under adversarial balance without discriminator collapse. The risk of overfitting is reduced through the use of early stopping and learning-rate scheduling, which help stabilize training for both the generator and discriminator.

A practical hyperparameter schedule is adopted. Training is started with a relatively high learning rate (0.0001) and with no structural regularization (λ_spatial = 0, λ_angle = 0). Once plausible human shapes are observed—while residual limb twisting may still be present—regularization weights are gradually introduced and tuned (e.g., λ_spatial = 0.005, λ_angle = 0.005), and a small angular tolerance ε = 0.001 is set for the angle-consistency term. The learning rate is then reduced (e.g., to 0.00001) and training is continued. Early stopping is applied when generated poses deteriorate, after which the above hyperparameters are readjusted so that generalization is improved.

**Fig 8:**
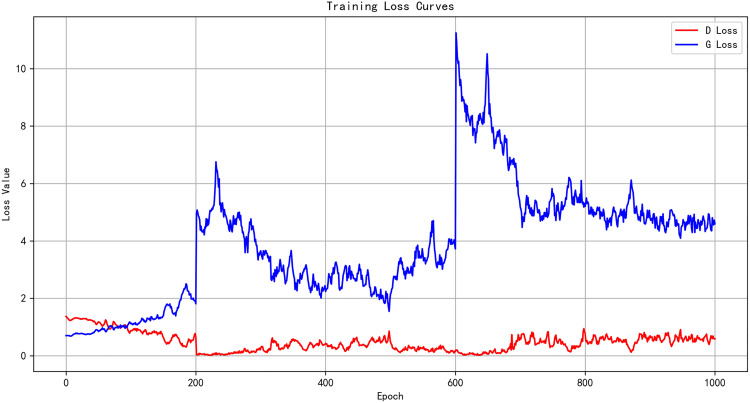
Training loss curves. (Blue: generator; Red: discriminator. X: epochs (0–1000); Y: loss value. The discriminator drops early and remains low, while the generator shows spikes near 200 and 600 before stabilizing—indicating a transition from early instability to a balanced adversarial regime without sustained mode collapse.).

The training curves in Fig 8 show that the generator loss (G Loss) and discriminator loss (D Loss) do not converge monotonically but instead oscillate within a stable range. This oscillatory behavior is typical in adversarial learning, reflecting the dynamic competition between the generator and discriminator. Over the course of training, both losses gradually reach a balance, indicating that the model has approached an equilibrium where the generator produces plausible samples and the discriminator can no longer easily distinguish real from generated data. Furthermore, the training strategy first performs unconstrained learning until the generated poses visually resemble human-like structures. At this stage, constraint terms are incorporated to guide and refine the generated data, ensuring both structural plausibility and stability. To prevent overfitting during training, early stopping and learning rate adjustment techniques are applied. These strategies help maintain generalization by halting training when the model’s performance levels off. Additionally, the learning rate is gradually lowered to facilitate fine-tuning. As a result, the generator’s training curve may exhibit sudden upward spikes, reflecting the model’s adaptation to these constraints and fine-tuning processes.

**Fig 9:**
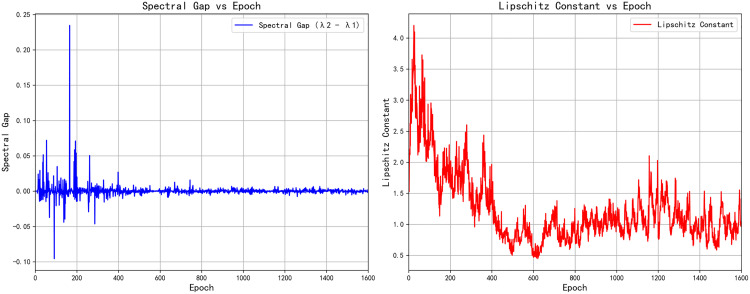
Spectral gap (left) and Lipschitz constant (right) monitoring in adversarial training.

The Fig 9 displays the variation of the spectral gap and Lipschitz constant with respect to Epochs during adversarial training. Both metrics gradually stabilize during the training process, reflecting the convergence of the model’s training.

In the Fig 9 as training progresses, the values of spectral gap and Lipschitz constant show a trend of stabilization. This indicates that the min-max game between the generator and discriminator gradually reaches equilibrium. Specifically, the smaller fluctuations in spectral gap and Lipschitz constant after a certain number of epochs suggest that the model is stabilizing, and the training process is nearing convergence. The stabilization of the spectral gap reflects the steadying of the graph structure and the internal information flow of the network. This suggests that the interaction between the generator and discriminator is becoming balanced, and the quality of the model’s output is continuously improving. The stabilization of the Lipschitz constant indicates that the model’s parameter updates and training process are becoming more stable, avoiding issues like gradient explosion or excessive updates, further validating the stability and convergence of the training process.

The spectral gap refers to the difference between the eigenvalues λ2 and λ1 of the graph, which reflects the connectivity and stability of the graph. In adversarial training, a larger spectral gap means a greater difference between the generator and discriminator, leading to instability in the network’s training. Conversely, a smaller spectral gap indicates that the generator and discriminator’s training is aligning, and the model is converging more effectively. Therefore, monitoring the spectral gap helps in observing the stability of the model. The Lipschitz constant measures the degree to which the output of a function changes with respect to changes in its input. In deep learning, a smaller Lipschitz constant typically indicates that the model is less sensitive to input changes, resulting in smoother gradient updates. A larger Lipschitz constant, on the other hand, can lead to gradient explosion or unstable training. In adversarial training, the stabilization and smaller value of the Lipschitz constant mean that the training process is more stable, avoiding large parameter updates, which in turn enhances convergence efficiency.

By introducing the DynamicGraphGNN operation and the spatial constraints regularization, the training process of the min-max adversarial game can achieve effective equilibrium, avoiding instability and non-convergence issues during training. The theoretical foundation of this approach is based on the concept of Nash equilibrium, combined with the spectral properties and Lipschitz continuity found in modern Graph Neural Networks (GNNs). Ultimately, the generator and discriminator will gradually approach equilibrium during the training process, leading to the stable convergence of the model.

### 4.4. Evaluation model for generated data

Traditional image quality evaluation metrics such as PSNR [33] and FID [34] are reference-based and primarily measure the pixel-level or distributional similarity between the generated image and the original image. As the generated data becomes increasingly similar to the ground truth, these metrics tend to improve monotonically in a single direction. However, in the context of generative adversarial networks (GANs), the goal is to create realistic samples that match the training data in style and distribution, rather than replicating the original images pixel by pixel. Unlike image synthesis, our task generates 3D skeletal joint coordinates (17 × 3) rather than pixel images. Image-centric metrics such as PSNR (higher is better) and FID (lower is better) primarily reflect pixel-space appearance and are highly sensitive to rendering choices (e.g., line width, color, camera pose, etc.), while being largely insensitive to kinematic/physiological plausibility (bone-length consistency, joint-angle limits, left–right symmetry, etc.). Consequently, it is possible for visually incorrect or semantically meaningless images to achieve comparable PSNR or FID scores, highlighting a key limitation of traditional metrics. Therefore, this article proposes a novel evaluation metric specifically designed to assess the quality of 3D skeletal samples generated by generative adversarial networks.

After the CDGSPAN model generates 3D skeletal samples, it is necessary to evaluate whether the generated sample is similar to the real training samples and whether it exhibits valid human motion characteristics—such as continuity of joint movement, reasonable limb lengths, and physiological feasibility. Inspired by MPJPE [32], a new evaluation metric is proposed to assess the quality of generated 3D skeletal samples. Let the generated 3D pose sequence be represented as $K_{\text{G}}=[f_{\text{1}},...,f_{k},...,f_{B}]\in {\text{R}}^{B\times 17\times 3},f_{k}\in {\text{R}}^{17\times 3}$. The corresponding ground-truth pose sequence is denoted as $K=[z_{\text{1}},...,z_{k},...,z_{B}]\in {\text{R}}^{B\times 17\times 3},\;z_{k}\in {\text{R}}^{17\times 3}$. The mean error $\overset{―}{e}$ and standard deviation $\sigma_{e}$ between corresponding joints of generated and real poses are computed as follows:


$$\overset{―}{e}=\frac{\text{1}}{17B}\sum_{k=1}^{B}\sum_{i=1}^{17}∥f_{k}\left(i\right)-z_{mean}\text{(}i\text{)}∥,z_{mean}=\frac{\text{1}}{B}\sum_{k=1}^{B}z_{k}\in {\text{R}}^{17\times 3}$$ (17)

$$\sigma_{e}=\sqrt{\frac{\text{1}}{17\cdot B}\sum_{k=1}^{B}\sum_{i=1}^{17}(∥f_{k}(i)-z_{mean}\text{(}\text{i}\text{)}∥-\overset{―}{e}{)}^{\text{2}}}$$ (18)

Subsequently, for both the generated and ground-truth 3D skeletal sequences, joint triplets (groups of three joints) are sampled. These triplets are substituted into Equations (9) through (12) to compute the angular similarity metric $L_{\text{angle}}$, which measures the deviation in joint angles. Then, the binary cross-entropy (BCE) loss between the generated sequence and the real sequence is computed using the discriminator $L_{\text{disc}}=\sum_{k=1}^{B}{max}_{c\in \{1,\ldots ,C\}}\text{softmax}(D^{\prime }(K_{G}[k,:,:]){)}_{c}$, This loss quantifies how well the generated sample is mistaken for a real one by the new discriminator *D’* The newly trained discriminator *D’* adopts the same architecture as shown in Fig 6 and is trained on the same dataset as reported in Table 2,which is used to evaluate the generated samples. It achieves accuracies of 99.51% on the training set, 99.48% on the validation set, and 99.39% on the test set. Finally, a comprehensive loss score is computed as:


$$t_{\text{score}}=(L_{\text{disc}}{)}^{\text{2}}+(L_{\text{angle}}{)}^{\text{2}}+{\overset{―}{e}}^{\text{2}}+\sigma_{e}^{\text{2}}$$ (19)

Equation (19) serves as an integrated metric to evaluate the generated pose sequence in terms of reconstruction error, positional deviation, angular deviation, and adversarial loss. A lower *t*_score_ indicates that the 3D pose sequence produced by the CDGSPAN model is more similar to the ground-truth sequence. Conversely, if the generated poses contain noise, or the spatial configuration of joints violates human motion constraints, the corresponding *t*_score_ will be significantly higher.

### 4.5. Style-specific sample selection

To determine whether a generated pose corresponds to a specific style, we use a reference skeleton $𝐐\in {\mathbb{R}}^{17\times 3}$ that characterizes the target style. Each generated candidate ${𝐆}_{i}\in {\mathbb{R}}^{17\times 3},i=1,\ldots ,N$, is evaluated against this reference using the Frobenius norm:


$$d\left({𝐆}_{i},𝐐\right)=\|{𝐆}_{i}-{𝐐\|}_{F}=\sqrt{\sum_{j=1}^{17}\sum_{k=1}^{\text{3}}{\left({𝐆}_{i}\left(j,k\right)-𝐐\left(j,k\right)\right)}^{\text{2}}}$$ (20)

A tolerance margin τ>0 is introduced to account for natural variability. The set of candidates that meet this condition is given by


$${𝒮}_{\tau }=\left\{i|d\left({𝐆}_{i},𝐐\right)\leq \tau \right\}$$ (21)

All candidates in ${𝒮}_{\tau }$ can be regarded as valid realizations of the target style, since their deviations from the reference skeleton remain within the acceptable margin. Among these, the most representative sample i^*^ is identified as the one with the minimum error:


$$i^{\text{*}}=\text{arg}\underset{i\in {𝒮}_{\tau }}{\text{min}}\;d\left({𝐆}_{i},𝐐\right)$$ (22)

If no candidate satisfies the tolerance, the threshold τ may be relaxed, or the top-k closest samples may be retained. This procedure ensures that generated poses are filtered and selected strictly according to their proximity to the designated style, thereby providing a consistent criterion for style-constrained pose generation.

## 5. Experiments and evaluation

### 5.1. Comparative evaluation with other models

Adversarial models for skeleton generation are proposed by many researchers in recent years. However, most of these models are trained on high-resolution datasets containing densely annotated data (such as image collections with over 100,000 samples), where a large amount of structural information is extracted from pixel-level features. As a result, such models are not designed to capture sparse spatial structures or joint-level relationships. Therefore, in this study, a sparse 17 × 3 skeletal representation is adopted, and comparative experiments are conducted using representative adversarial models, including MLPGAN [35], GRAGAN [36], SAGAN [37], DCGAN [38], CNNGAN [39] and RESNETGAN [40]. CNNGAN and RESNETGAN adopt conventional CNN and RESNET architectures for feature extraction within their adversarial framework. The evaluation metric is defined by Equation (19), and the results are shown in Table 4. CDGSPAN achieves the lowest *t*_score_, and the generated samples are closer to the real samples than those produced by other models. The lowest mean error $\overset{―}{e}$ and standard deviation $\sigma_{e}$ are observed. As shown in Table 4, CDGSPAN achieves a discriminator $L_{\text{disc}}$ loss of 7.1205, which is in lower level. This indicates that the samples generated by CDGSPAN are more difficult to distinguish from real samples by the discriminator. Meanwhile, it is worth noting that the tested models such as DCGAN, CNNGAN, and RESNETGAN exhibit lower values in certain traditional loss metrics (e.g., $L_{\text{disc}}$) compared to the proposed CDGSPAN.

**Table 4:** Evaluation metrics for different methods.

Method	*L*_disc_	*L*_angle_(rad)	$\overset{―}{e}$ (m)	$\sigma_{e}$ (m)	*t*_score_	Model complexity (parameters)	Average inference time (100 trals) (s)
MLPGAN	7.4842	83.0964	0.4678	0.2119	6961.2944	0.1341M	3.3635E-04
GRAGAN	8.0000	65.2723	0.7493	0.2971	4325.1211	0.0030M	2.8270E-04
SAGANGAN	7.8359	86.4269	0.5425	0.2013	7531.3428	0.0182M	5.8260E-04
DCGAN	7.4296	46.7649	0.2796	0.1441	2242.2576	0.0344M	4.6291E-04
CNNGAN	7.5032	69.1314	0.5427	0.3051	4835.8330	0.0134M	3.8835E-04
RESNETGAN	7.9917	72.5344	0.4937	0.2541	5325.4180	0.0132M	2.9536E-04
CDGSPAN	**7.1205**	**21.6705**	**0.1494**	**0.0918**	**520.3430**	**0.0272M**	**8.1454E-04**

According to the data in Table 4, although CDGSPAN has a lower discriminator’s confidence (*L*_disc_ = 7.1205) compared to other methods (e.g., DCGAN with *L*_disc_ = 7.4296), it decisively outperforms all baselines on domain-relevant criteria. Specifically, CDGSPAN’s joint-angle penalty (*L*_angle_) drops to 21.6705 rad, a reduction of about 74% compared to other methods (e.g., MLPGAN with *L*_angle_ = 83.0964 rad), indicating significantly fewer extreme or illegal angles in the generated 3D skeletons. In terms of geometric accuracy and stability, CDGSPAN’s mean error (*ē*) is 0.1494, which is much lower than other methods (e.g., RESNETGAN with *ē* = 0.4937), demonstrating better accuracy. Furthermore, its standard deviation (*σ*_*e*_) is 0.0918, significantly lower than MLPGAN and SAGAN, indicating that CDGSPAN produces more consistent and stable poses. In the task-specific quality metric, *t*_score_, CDGSPAN achieves a value of 520.3430, much lower than other methods, such as CNNGAN with *t*_score_ = 4835.8330. This shows that CDGSPAN delivers superior structural plausibility, geometric consistency, and perceptual/physiological quality. Despite its higher model complexity (0.272M parameters) and inference time (8.1454E-04s), CDGSPAN consistently produces higher-quality results compared to other methods. Moreover, although CDGSPAN has a slightly longer inference time, it remains competitive in terms of efficiency, achieving high-quality results faster than most models. This highlights that, despite its higher complexity, CDGSPAN maintains a strong performance-to-efficiency ratio in generating high-quality 3D skeletons.

**Fig 10:**
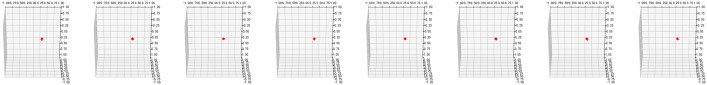
3D Skeleton sequence samples generated by the MLPGAN model.

From the comparison with ground-truth sample**s**, it is observed that the 3D skeleton sequences generated by CDGSPAN resemble the ground-truth ones more closely than the other methods. As illustrated in Figs 10–, the generated sequences closely match the structure of the ground-truth poses, with only minimal deviations. Although slight differences in limb motion patterns can be observed, the overall results exhibit strong consistency between the generated and real data. These visual observations align well with the quantitative results reported in Table 4, further confirming the effectiveness of CDGSPAN.

**Fig 11:**
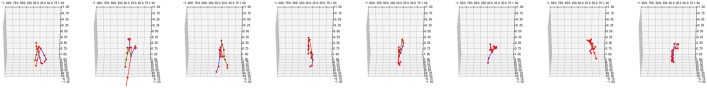
3D Skeleton sequence samples generated by the GRAGAN model.

**Fig 12:**
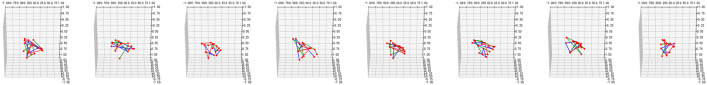
3D skeleton sequence samples generated by the SAGAN model.

**Fig 13:**
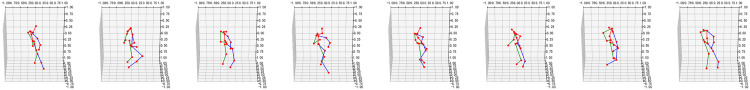
3D skeleton sequence samplesgenerated by the DCGAN model.

**Fig 14:**
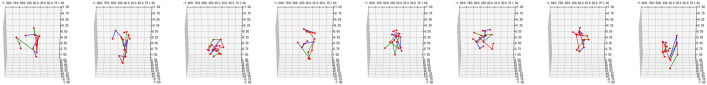
3D skeleton sequence samples generated by the CNNGAN model.

**Fig 15:**
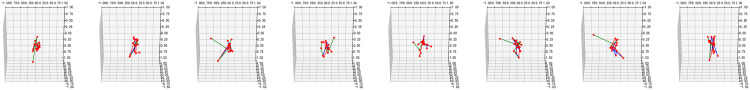
3D skeleton sequence samples generated by the RESNETGAN model.

**Fig 16:**
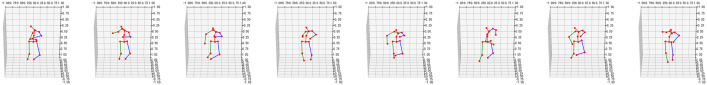
3D skeleton sequence samples generated by the CDGSPAN model. (CDGSPAN’s generated 3D skeleton sequences closely match the ground truth with only minor deviations, consistent with the quantitative results in Table 4.).

**Fig 17:**
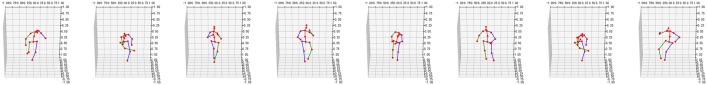
Ground-truth 3D skeleton sequence samples.

**Fig 18:**
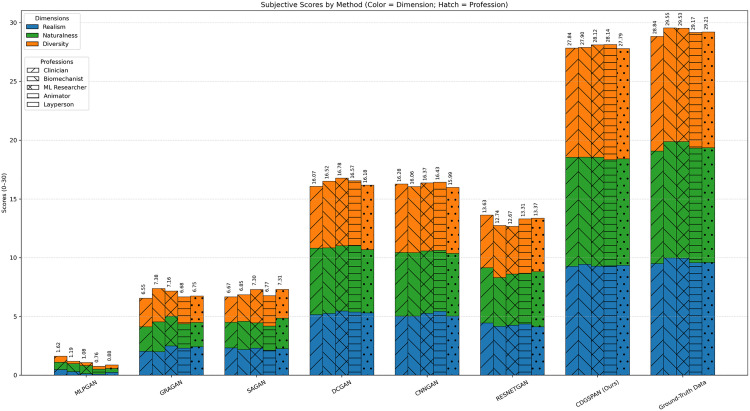
Subjective evaluation results of different GAN-based pose generation methods. (Across five rater groups, CDGSPAN nearly matches the ground truth (~28/30 vs. ~ 29/30 Overall) and consistently outperforms all baselines on Realism, Naturalness, and Diversity; the ranking (GT > CDGSPAN > others) is stable across professions.).

Fig 18 Stacked bar charts of mean subjective ratings per profession from five rater groups—cinician (n = 8), biomechanist (n = 6), ML (Machine Learning) researcher (n = 10), animator (n = 7), and layperson (n = 9); total *N* = 40—across eight conditions (MLPGAN, GRAGAN, SAGAN, DCGAN, CNNGAN, RESNETGAN, CDGSPAN (Ours), and ground-truth data). For each method, five side-by-side bars correspond to the five professions; each bar reflects the average score within that profession. Within every bar, colored segments denote Realism, Naturalness, and Diversity, and the total height gives the Overall score (0–30). Ground-truth serves as the perceptual upper bound (≈29/30), while CDGSPAN closely approaches this reference and visually outperforms the baselines on all three dimensions.

Across professions (group means), CDGSPAN attains Overall scores of 27.8–28.1, with mean gaps to ground truth of ~1.0–1.7 points (e.g., clinician 27.84 vs. 28.84, biomechanist 27.90 vs. 29.55, ML researcher 28.11 vs. 29.53, animator 28.14 vs. 29.17, layperson 27.79 vs. 29.21). Improved GAN baselines (DCGAN/CNNGAN/RESNETGAN) cluster around ~12–17, and traditional GANs (MLPGAN/GRAGAN/SAGAN) around ~1–7. The stack composition shows consistent gains in realism and naturalness (≈9.0–9.6 each) while maintaining strong diversity, and the ranking (ground truth> CDGSPAN> others) remains stable across all five profession-specific averages. The results are consistent with the *t*_score_ values reported in Table 4.

### 5.2. Ablation study

An ablation study is conducted to evaluate the impact of removing key components from the CDGSPAN model. As shown in Table 5, removing either key component leads to marked degradation. The full CDGSPAN achieves a joint-angle penalty of $L_{\text{angle}}=20.6016$ rad, which rises to 47.9018 rad (≈+132\%) without spatial constraints and to 25.9700 rad (≈+26\%) without the dynamic-graph operation. Geometric accuracy and stability follow the same trend: the full model’s $\overset{―}{e}=0.1449$ and $\sigma_{e}=0.0885$ deteriorate to 0.1898/0.1358(≈+31\%/+53\%) in the no-spatial variant and to 0.2377/0.1543(≈+64\%/+74\%) in the no-dynamic variant, showing both modules are crucial for suppressing implausible limb postures and improving kinematic consistency.

**Table 5:** Ablation test results.

Method	*L*_disc_	*L*_angle_ (rad)	$\overset{―}{e}$ (m)	$\sigma_{e}$ (m)	*t*_score_	Model complexity (parameters)	Average inference time (100 trals) (s)
Without spatial constraints	7.6261	47.9018	0.1898	0.1358	2352.7981	0.0272M	7.6006E-04
Without DynamicGraphGNN function (No dynamic graph operation)	7.2060	25.9700	0.2377	0.1543	726.4484	0.0264M	1.2549E-04
CDGSPAN (full model)	**7.4584**	**20.6016**	**0.1449**	**0.0885**	**480.0798**	**0.0272M**	**8.1265E-04**

Table 5 also shows that the composite quality metric *t*_score_, which captures perceptual and physiological plausibility, is most sensitive to these removals: the full model attains *t*_score_ = 480.0798; this balloons to 2352.7981(≈+389\%) without spatial constraints and 726.4484\ (≈+51\%) without the dynamic operation. Notably, discriminator confidence $L_{\text{disc}}$ alone can mislead: the no-dynamic model has $L_{\text{disc}}=7.2060$ (lower than the full model’s 7.4584), yet its $t_{\text{score}}$ is far worse—hence the need for $t_{\text{score}}$ as a task-relevant indicator.

Finally, Table 5 reports similar model sizes across settings (0.026−0.027M parameters) and inference times on the order of $10^{-4}$ s (full 8.1265E-04s; no-spatial 7.6006E-04s; no-dynamic 1.2549E-04s, faster after removing the dynamic operation). In sum, Table 5 demonstrates that without increasing model scale, the full CDGSPAN simultaneously achieves the lowest $L_{\text{angle}},\overset{―}{e},\sigma_{e}$, and $t_{\text{score}}$, confirming both the spatial-constraints and dynamic graph operation are indispensable for structural plausibility, kinematic consistency, and perceptual quality.

The ablation experiments above demonstrate that comprehensive loss score $t_{\text{score}}$ calculated using Equation (19) serves as a valid and reliable measure for evaluating generation quality.

**Fig 19:**
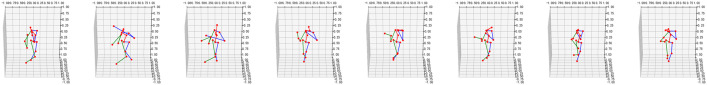
Samples generated without the spatial constraint module.

The 3D skeletal sequences generated from the models of Table 5 can be visually inspected and compared with the ground-truth samples as shown in Figs 19–. It can be observed that for the CDGSPAN models with the spatial constraint module or the dynamic graph operation removed, the generated poses exhibit mutual misalignment between limbs, and certain joint positions appear in anatomically implausible locations, resulting in a noticeably chaotic structure. In contrast, the full CDGSPAN model produces samples that resemble the ground-truth ones with no structural dislocation of limbs or joint positions that violate human motion kinematics in either spatial or angular dimensions.

**Fig 20:**
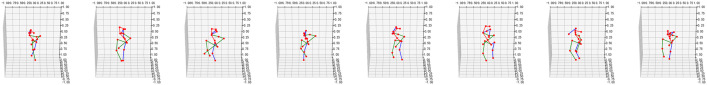
Samples generated without the dynamic graph operation.

**Fig 21:**
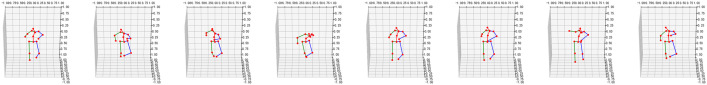
Samples generated by the full CDGSPAN model in the ablation study. (Removing the spatial constraint or motion regulation causes limb misalignment and anatomically implausible joints, whereas the full CDGSPAN matches the ground truth without kinematic violations; visuals align with Table 4 and support *t*_score_ Equation (19) as a reliable quality metric.).

**Fig 22:**
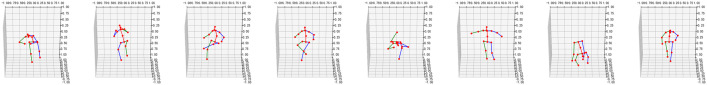
Ground-truth samples.

### 5.3. Evaluation of style-specific pose selection

**Fig 23:**
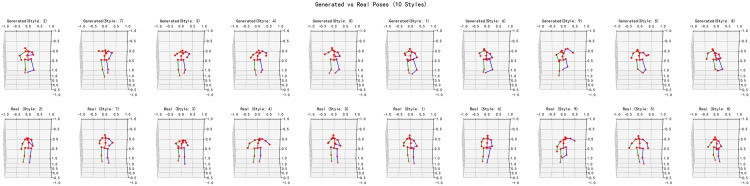
Style-specific pose generation. (Using nearest-neighbor matching over 17 × 3 keypoints, generated poses inherit style labels from their closest real exemplars; across 10 styles, the synthesized (top) closely align with matched real poses (bottom), preserving global topology with minor local deviations and confirming style diversity/alignment).

Building on the method of Section 4.5, we first sample poses from the proposed adversarial generator under random noise, then, for each generated sample, perform Euclidean nearest-neighbor as Equation (20), and match in the training or testing set over the full 17 × 3 keypoints representation to identify its closest real exemplar and inherit the corresponding style label. The Fig 23 arranges 10 distinct styles column-wise: the top row (Generated) displays the synthesized 3D skeletons, and the bottom row (Real) shows the matched real poses with their style annotations. Visually, generated poses preserve the global skeletal topology of their real counterparts—evidence that the model has learned plausible spatial/kinematic constraints—while minor local deviations (e.g., limb flexion, torso orientation) remain. The clear differentiation across columns confirms style diversity and indicates that Section 4.5’s selection and matching pipeline effectively ensures that generated samples are aligned with the correct style categories present in the training or testing samples.

**Fig 24:**
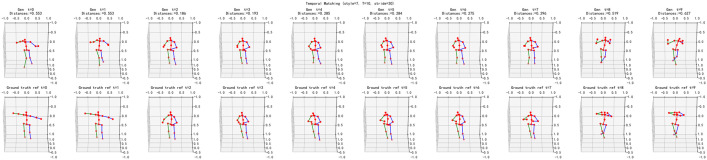
Temporally aligned pose generation. (Top row shows temporally coherent samples generated by the proposed model, while the bottom row shows real temporal samples that are used to guide the generation of the top sequence.).

A reference sequence of length T (here, T=10 frames) is used for temporal alignment. As shown in Fig 24, the reference is subsampled with an interval of 30 frames, meaning adjacent reference poses are taken 30 frames apart in the original stream. For each time step t, the distance displayed above the generated panel is computed using Equation (20); smaller values indicate closer matches. Following section 4.5, the real, temporally ordered sequence is employed to guide selection from a pool of model outputs: at each t, the candidate minimizing the distance in Eq. (20) is chosen, and a one-to-one constraint is enforced to prevent reuse. The selected poses are then concatenated over time, by which a temporally aligned, model-generated sequence is obtained that mirrors the progression of the real sequence while preserving the target style.

### 5.4. Visual sensitivity analysis of constraint loss weights

For the parameter sensitivity test, λ_spatial = 0.005 and λ_angle = 0.005 are used as the baseline configuration. The two parameters are adjusted individually and in combination, and the generated samples under these settings are visually compared to assess the effect of the constraint weights. It is observed that when λ_spatial or λ_angle is set too large or too small, the generated 3D skeletons become distorted, for example, sample diversity is suppressed, and limb lengths deviate from normal human proportions, while moderate values (λ_spatial = 0.005 and λ_angle = 0.005) lead to more natural and diverse results. The sensitivity test results are shown in –. Relative to the baseline (λ_spatial = 0.005, λ_angle = 0.005), increasing or decreasing either weight—alone or together—yields distorted 3D skeletons (reduced diversity, abnormal limb lengths), whereas the baseline produces the most natural and diverse samples.

**Fig 25:**
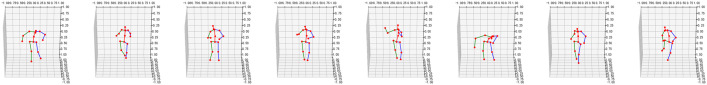
Generated samples with λ_spatial = 0.005 and λ_angle = 0.005.

**Fig 26:**
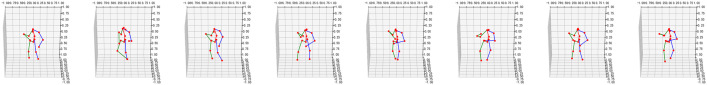
Generated samples with λ_spatial = 0.005 and λ_angle = 0.0005.

**Fig 27:**
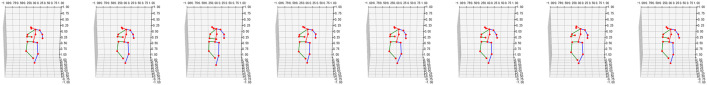
Generated samples with λ_spatial = 0.005 and λ_angle = 0.025.

**Fig 28:**
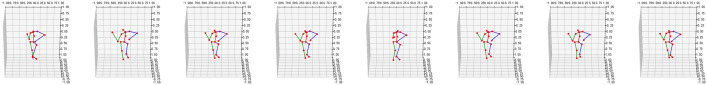
Generated samples with λ_spatial = 0.0005 and λ_angle = 0.005.

**Fig 29:**
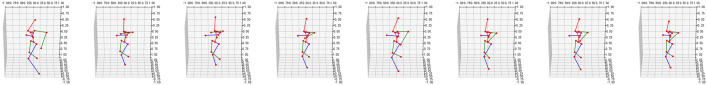
Generated samples with λ_spatial = 0.025 and λ_angle = 0.005.

### 5.5. Statistical evaluation of CDGSPAN performance

**Fig 30:**
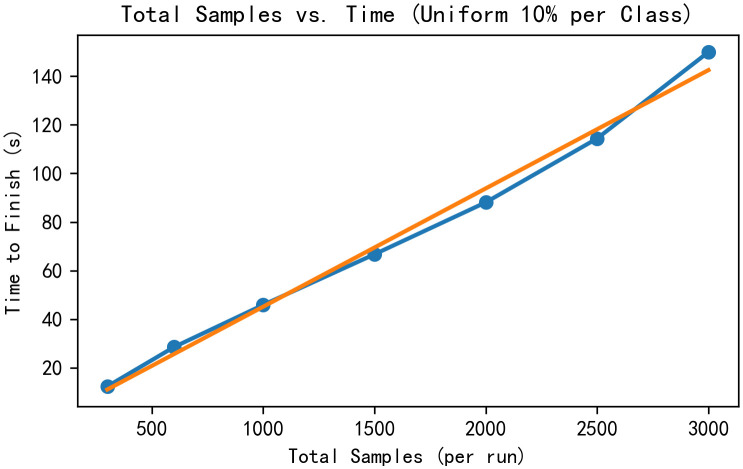
Total samples vs. time.

**Fig 31:**
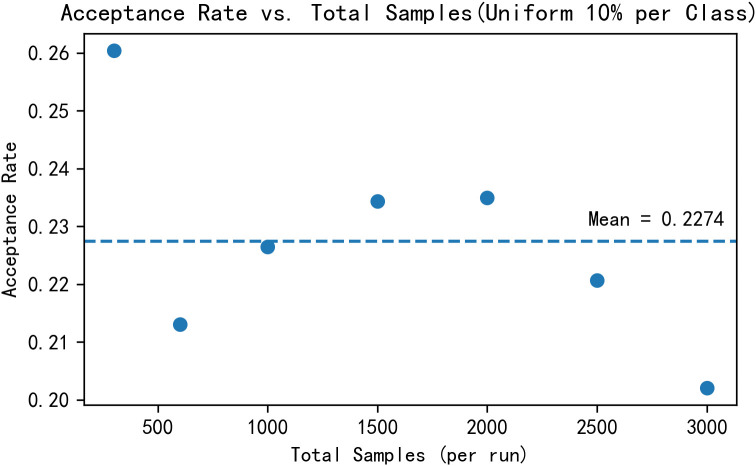
Total samples vs. acceptance rate.

We evaluated sampling efficiency and acceptance‐rate stability under a uniform per-class quota. Using the full training set (per-frame 17 × 3 keypoints), poses were normalized by centering on the pelvis/root joint and scaling by the mean joint-to-root distance. A pretrained generator produced samples from standard-normal noise, and each candidate was assigned a class via Euclidean nearest neighbor to the normalized training set. For total sample sizes *N*_sample_∈{300,600,1000,1500,2000,2500,3000} as shown in the Figs 30 and 31, we enforced a 10% target per class (or uniform 1/*C* for 10 different style categories) and stopped once all quotas were met. We recorded wall-clock time to completion and defined acceptance rate as the ratio of accepted samples to all evaluated candidates. The figures report total samples vs. time with an ordinary least-squares fit (slope and *R*^2^) and total samples vs. acceptance rate with an in-figure dashed mean line; all measurements are collected on the same hardware/software configuration for consistency.

Under a uniform 10% per-class quota, time-to-quota increases approximately linearly with total samples and the acceptance rate remains stable across scales. No collection bottlenecks or declines in acceptance are observed, indicating no evident performance impact from class-wise dataset bias and any residual effect, if present, is small and does not manifest as measurable efficiency or acceptance rate degradation. Overall, the proposed method (CDGSPAN) shows good scalability and robustness within the current data distribution.

The model’s generalization can be affected if the 3D skeleton samples use inconsistent coordinate conventions. If other videos or images are reconstructed and then mapped to the same coordinate system as the training data—i.e., scaled and normalized so that keypoint coordinates are unified—then even a single-source dataset can train a well-generalizing model. When training on multiple datasets, it is essential to standardize the 3D skeleton keypoint coordinates across datasets. Therefore, a robust preprocessing pipeline must be established to perform the necessary coordinate alignment and normalization.

Across ten independent runs per method (Trials = 10), the composite score $t_{\text{score}}={\left(L_{\text{disc}}\right)}^{\text{2}}+{\left(L_{\text{angle}}\right)}^{\text{2}}+{\overset{―}{e}}^{\text{2}}+\sigma_{e}^{\text{2}}$ consistently ranks methods in a way that matches our qualitative, visual assessment of the generated 3D skeletons. Smaller $t_{\text{score}}$ indicates better sample quality. In the Table 6, CDGSPAN yields the lowest $t_{\text{score}}$ values run-by-run, while baselines such as CNNGAN, DCGAN, GraphGAN, MLPGAN, RESNETGAN, and SAGAN exhibit substantially larger scores. This behavior is stable across runs and reflects the constituent terms as well. CDGSPAN maintains lower $L_{\text{angle}}$, $L_{\text{disc}}$, ${\overset{―}{e}}^{\text{2}}\;and\;\sigma_{e}^{\text{2}}$, which is resulting in markedly reduced $t_{\text{score}}$. Overall, the numerical results corroborate the visual inspection of generated poses. Methods with lower $t_{\text{score}}$ also produce visibly cleaner, more anatomically plausible 3D skeleton samples.

**Table 6:** Run-level raw metrics and composite tscore by method (lower is better).

Run_id	Method	Trials	*L*_disc_	*L*_angle_(rad)	$\overset{―}{e}$ (m)	$\sigma_{e}$ (m)	*t*_score_
**0**	CDGSPAN	10	**8.9349**	**24.1748**	**0.1440**	**0.0989**	**664.2863**
**0**	CNNGAN	10	9.9979	87.9154	0.5301	0.3054	7829.4470
**0**	DCGAN	10	9.9776	55.8582	0.2743	0.1363	3219.7850
**0**	GraphGAN	10	10.0000	76.0092	0.6355	0.2328	5877.8600
**0**	MLPGAN	10	8.7501	104.5034	0.4686	0.2097	10997.7800
**0**	RESNETGAN	10	10.0000	95.1265	0.5120	0.2487	9149.3720
**0**	SAGAN	10	9.5143	105.5323	0.5388	0.1982	11227.9100
**1**	CDGSPAN	10	**8.9608**	**25.6977**	**0.1414**	**0.0892**	**740.6971**
**1**	CNNGAN	10	9.9926	91.1071	0.5492	0.3016	8400.7550
**1**	DCGAN	10	9.4136	57.9670	0.2911	0.1639	3448.8980
**1**	GraphGAN	10	9.9998	66.5857	0.6635	0.2316	4534.1440
**1**	MLPGAN	10	8.4119	99.5908	0.4680	0.2086	9989.3450
**1**	RESNETGAN	10	10.0000	97.0141	0.5160	0.2590	9512.0650
**1**	SAGAN	10	9.9284	102.7262	0.5352	0.2033	10651.5800
**2**	CDGSPAN	10	**7.9983**	**26.0658**	**0.1316**	**0.0851**	**743.4220**
**2**	CNNGAN	10	9.9992	83.9414	0.5079	0.2928	7146.4780
**2**	DCGAN	10	8.9227	56.7820	0.2704	0.1509	3303.9070
**2**	GraphGAN	10	10.0000	74.2851	0.6695	0.2240	5618.7770
**2**	MLPGAN	10	8.5537	111.0584	0.4696	0.2107	12407.4000
**2**	RESNETGAN	10	9.9021	94.7738	0.4918	0.2604	9080.4370
**2**	SAGAN	10	9.0422	114.1821	0.5470	0.2114	13119.6600
**3**	CDGSPAN	10	**8.2054**	**30.7402**	**0.1349**	**0.0866**	**1012.3150**
**3**	CNNGAN	10	9.5987	87.3787	0.4976	0.2802	7727.4920
**3**	DCGAN	10	9.7006	66.9399	0.2660	0.1496	4575.1460
**3**	GraphGAN	10	10.0000	86.2104	0.6660	0.2276	7532.7290
**3**	MLPGAN	10	8.3815	106.0974	0.4690	0.2107	11327.1600
**3**	RESNETGAN	10	9.9619	96.3104	0.4818	0.2377	9375.2250
**3**	SAGAN	10	9.9721	100.1814	0.5444	0.2094	10136.1000
**4**	CDGSPAN	10	**7.7338**	**24.7292**	**0.1453**	**0.1014**	**671.3757**
**4**	CNNGAN	10	9.9894	96.1787	0.5559	0.3242	9350.5420
**4**	DCGAN	10	9.7259	57.6192	0.2696	0.1354	3414.6530
**4**	GraphGAN	10	9.9999	68.2132	0.6686	0.2314	4753.5330
**4**	MLPGAN	10	9.3583	105.5229	0.4684	0.2121	11222.9200
**4**	RESNETGAN	10	9.9079	93.5619	0.4849	0.2283	8852.2890
**4**	SAGAN	10	9.9979	98.4018	0.5456	0.1970	9783.2010
**5**	CDGSPAN	10	**8.1412**	**22.1860**	**0.1470**	**0.0833**	**558.5269**
**5**	CNNGAN	10	9.8980	102.1487	0.5080	0.3037	10532.6700
**5**	DCGAN	10	9.7750	57.3426	0.2780	0.1357	3383.8190
**5**	GraphGAN	10	10.0000	76.9110	0.6440	0.2230	6015.7660
**5**	MLPGAN	10	8.5374	107.7776	0.4698	0.2096	11689.1600
**5**	RESNETGAN	10	9.9879	96.3018	0.5343	0.2448	9374.1420
**5**	SAGAN	10	9.9992	100.1670	0.5453	0.2196	10133.7500
**6**	CDGSPAN	10	**8.7711**	**16.2920**	**0.1117**	**0.0643**	**342.3771**
**6**	CNNGAN	10	9.9884	93.4509	0.5093	0.3139	8833.1880
**6**	DCGAN	10	9.9946	52.4952	0.2911	0.1447	2855.7420
**6**	GraphGAN	10	9.5332	70.1057	0.6657	0.2349	5006.1830
**6**	MLPGAN	10	8.4681	100.4443	0.4679	0.2111	10161.0400
**6**	RESNETGAN	10	9.9997	104.9852	0.5140	0.2493	11122.2000
**6**	SAGAN	10	9.2213	109.7927	0.5523	0.2067	12139.8200
**7**	CDGSPAN	10	**8.3848**	**27.4484**	**0.1634**	**0.1273**	**823.7642**
**7**	CNNGAN	10	9.9179	87.7861	0.5469	0.3096	7805.1510
**7**	DCGAN	10	9.8683	69.9056	0.2646	0.1430	4984.2680
**7**	GraphGAN	10	10.0000	75.8043	0.7194	0.2438	5846.8720
**7**	MLPGAN	10	9.4791	100.1847	0.4686	0.2102	10127.0800
**7**	RESNETGAN	10	10.0000	100.5397	0.4936	0.2547	10208.5400
**7**	SAGAN	10	9.9552	99.2542	0.5519	0.2085	9950.8440
**8**	CDGSPAN	10	**8.3163**	**20.6547**	**0.1650**	**0.1331**	**495.8210**
**8**	CNNGAN	10	9.9995	81.1955	0.5056	0.3012	6693.0440
**8**	DCGAN	10	9.8238	61.9717	0.2869	0.1664	3937.1130
**8**	GraphGAN	10	9.9194	76.8139	0.6671	0.2293	5999.2720
**8**	MLPGAN	10	8.0175	104.9944	0.4686	0.2101	11088.3700
**8**	RESNETGAN	10	10.0000	82.7897	0.4897	0.2088	6954.4120
**8**	SAGAN	10	9.9950	105.8804	0.5326	0.2043	11310.8900
**9**	CDGSPAN	10	**9.4790**	**27.2695**	**0.1424**	**0.0831**	**833.5036**
**9**	CNNGAN	10	9.7656	86.3128	0.5722	0.2973	7545.6750
**9**	DCGAN	10	9.7134	62.9154	0.2764	0.1539	4052.7950
**9**	GraphGAN	10	10.0000	79.9948	0.6856	0.2370	6499.6940
**9**	MLPGAN	10	8.5406	99.4136	0.4691	0.2111	9956.2760
**9**	RESNETGAN	10	10.0000	94.3988	0.4807	0.2304	9011.4210
**9**	SAGAN	10	10.0000	96.7151	0.5267	0.2003	9454.1360

The Table 7 reports repeat-aligned comparisons on the raw *t*_score_*A*_ (lower is better; smaller values indicate the generated 3D skeletons are closer to real samples). For each pair (A,B) we summarize the paired mean difference *d = t*_score_*A*_ *– t*_score_*B*_ together with its 95% confidence intervals (paired-t and bootstrap), a one-sided Wilcoxon signed-rank p-value for d>0, and the Hodges–Lehmann (HL) median difference with its bootstrap CI. A clear and consistent pattern emerges: whenever *B* = CDGSPAN, the mean difference is large in magnitude and negative (e.g., CDGSPAN vs. CNNGAN: −7497.8400; vs. DCGAN: −3029.0040; vs. MLPGAN: −10208.0400, etc.). Because d<0 means A’s tscore is larger, these rows show that CDGSPAN attains the smaller (better) *t*_score_ in every pairing. Moreover, for all “CDGSPAN vs. baseline” rows, both the paired-t and the bootstrap 95% CIs lie entirely below zero (e.g., CDGSPAN vs. CNNGAN: t-CI [−8358.8200, −6636.8500], boot-CI [−8236.7600, −6858.3600]), and intervals excluding 0 provide strong evidence that the observed differences are not due *t*o sampling variation.

**Table 7:** Pairwise (repeat-aligned) comparisons on raw *tscore* (lower is better). (Columns: A_model/B_model = methods compared; n_pairs(ℐ) = number of paired runs; mean_diff (*A*-*B*) = ${\overset{―}{\Delta }}_{A-B}=\frac{\text{1}}{n}\sum_{i\in ℐ}\left({\text{tscore}}_{A,i}-{\text{tscore}}_{B,i}\right)$ (<0→ *A* has a smaller/better tscore, if <0, *B* is better, otherwise, no difference.); t_CI_low/high = 95% paired-t CI for the mean difference; boot_CI_low/high = 95% bootstrap CI for the mean difference; wilcoxon_p (one-sided A-B > 0) = one-sided Wilcoxon signed-rank p-value (α = 0.05); HL_median = Hodges-Lehmann median difference; HL_boot_CI_low/high = bootstrap 95% CI for HL.).

A_model	B_model	n_pairs	mean_diff(*A*-*B*)	t_CI_low	t_CI_high	boot_CI_low	boot_CI_high		wilcoxon_p (one-sided A-B > 0) (α = 0.05)	HL_median	HL_boot_CI_low	HL_boot_CI_high
CDGSPAN	CNNGAN	10	**−7497.8400**	**−8358.8200**	**−6636.8500**	**−8236.7600**	**−6858.3600**	**1.0000**		**−7073.2700**	**−8490.8100**	**−6559.1200**
CDGSPAN	DCGAN	10	**−3029.0000**	**−3421.3400**	**−2636.6700**	**−3368.8000**	**−2731.8700**	**1.0000**		**−2784.2900**	**−3441.2900**	**−2560.4900**
CDGSPAN	GraphGAN	10	**−5079.8700**	**−5645.5200**	**−4514.2300**	**−5544.2000**	**−4607.6400**	**1.0000**		**−5118.3400**	**−5561.7100**	**−4478.7600**
CDGSPAN	MLPGAN	10	**−10208.0000**	**−10808.1000**	**−9608.0000**	**−10710.1000**	**−9712.3600**	**1.0000**		**−10324.2000**	**−10841.1000**	**−9303.3200**
CDGSPAN	RESNETGAN	10	**−8575.4000**	**−9348.6400**	**−7802.1600**	**−9219.1000**	**−7933.4300**	**1.0000**		**−8424.0000**	**−9078.0700**	**−8180.9100**
CDGSPAN	SAGAN	10	**−10102.2000**	**−10999.6000**	**−9204.7500**	**−10864.8000**	**−9410.7900**	**1.0000**		**−9743.0600**	**−10854.2000**	**−9119.4500**
CNNGAN	CDGSPAN	10	**7497.8350**	**6636.8500**	**8358.8200**	**6840.8930**	**8249.0200**	**0.0010**		**7073.2740**	**6559.1160**	**8490.8110**
CNNGAN	DCGAN	10	4468.8310	3386.7880	5550.8740	3613.9220	5374.4760	0.0010		4226.1160	3152.3460	5935.8890
CNNGAN	GraphGAN	10	2417.9610	1243.5510	3592.3710	1465.7250	3396.8440	0.0010		1954.9330	1045.9810	4171.9550
CNNGAN	MLPGAN	10	−2710.2100	−3689.6700	−1730.7500	−3546.5500	−1938.9000	1.0000		−2366.2700	−3781.8300	−1588.5900
CNNGAN	RESNETGAN	10	−1077.5700	−1925.4600	−229.6690	−1732.5500	−350.0920	0.9902		−1392.8400	−1968.3700	−261.3680
CNNGAN	SAGAN	10	−2604.3400	−3934.5700	−1274.1100	−3720.7100	−1523.7700	0.9990		−2329.7100	−3962.2400	−1289.1800
DCGAN	CDGSPAN	10	**3029.0040**	**2636.6710**	**3421.3360**	**2739.1580**	**3372.1600**	**0.0010**		**2784.2850**	**2560.4850**	**3441.2920**
DCGAN	CNNGAN	10	−4468.8300	−5550.8700	−3386.7900	−5365.3700	−3618.9400	1.0000		−4226.1200	−5935.8900	−3152.3500
DCGAN	GraphGAN	10	−2050.8700	−2563.2000	−1538.5400	−2447.3000	−1619.1900	1.0000		−2232.6600	−2631.9500	−1338.8800
DCGAN	MLPGAN	10	−7179.0400	−8008.7300	−6349.3500	−7836.4500	−6486.7100	1.0000		−7228.2800	−8041.6700	−6327.7500
DCGAN	RESNETGAN	10	−5546.4000	−6484.4900	−4608.3100	−6324.7500	−4774.2600	1.0000		−5607.0800	−5990.3200	−4958.6300
DCGAN	SAGAN	10	−7073.1800	−8229.4000	−5916.9500	−8046.0900	−6136.5500	1.0000		−6976.3100	−8328.9200	−5560.9500
GraphGAN	CDGSPAN	10	**5079.8740**	**4514.2280**	**5645.5200**	**4621.6580**	**5532.1170**	**0.0010**		**5118.3410**	**4552.6320**	**5561.7140**
GraphGAN	CNNGAN	10	−2417.9600	−3592.3700	−1243.5500	−3393.0000	−1456.0000	1.0000		−1954.9300	−4171.9600	−1045.9800
GraphGAN	DCGAN	10	2050.8700	1538.5370	2563.2040	1611.9910	2447.6020	0.0010		2232.6550	1338.8790	2631.9460
GraphGAN	MLPGAN	10	−5128.1700	−5891.7600	−4364.5800	−5755.3800	−4495.9300	1.0000		−5137.3900	−5962.2900	−4280.2100
GraphGAN	RESNETGAN	10	−3495.5300	−4572.6000	−2418.4600	−4382.1400	−2591.8800	1.0000		−3410.0200	−4538.3400	−2511.7300
GraphGAN	SAGAN	10	−5022.3100	−6185.1100	−3859.5000	−5981.5600	−4101.4800	1.0000		−5170.6400	−6241.8400	−3536.2200
MLPGAN	CDGSPAN	10	**10208.0400**	**9608.0010**	**10808.0900**	**9732.2950**	**10709.7200**	**0.0010**		**10324.1700**	**9303.3200**	**10841.0900**
MLPGAN	CNNGAN	10	2710.2090	1730.7450	3689.6720	1949.9540	3554.6860	0.0010		2366.2670	1588.5900	3781.8310
MLPGAN	DCGAN	10	7179.0400	6349.3470	8008.7330	6492.0670	7847.4100	0.0010		7228.2760	6327.7480	8041.6690
MLPGAN	GraphGAN	10	5128.1700	4364.5800	5891.7600	4507.3010	5765.4910	0.0010		5137.3870	4280.2120	5962.2920
MLPGAN	RESNETGAN	10	1632.6420	520.3194	2744.9650	722.7705	2545.6100	0.0098		1900.1720	431.6982	2820.9920
MLPGAN	SAGAN	10	105.8642	−686.6350	898.3637	−563.7340	755.3585	0.4609		−23.1387	−662.2350	1191.0640
RESNETGAN	CDGSPAN	10	**8575.4020**	**7802.1640**	**9348.6400**	**7947.6190**	**9220.6020**	**0.0010**		**8423.9980**	**8180.9130**	**9078.0740**
RESNETGAN	CNNGAN	10	1077.5670	229.6689	1925.4650	354.2954	1728.1610	0.0137		1392.8350	261.3682	1933.9590
RESNETGAN	DCGAN	10	5546.3980	4608.3050	6484.4910	4770.9560	6322.1900	0.0010		5607.0830	4958.6260	5996.3770
RESNETGAN	GraphGAN	10	3495.5280	2418.4560	4572.6000	2606.6260	4380.7740	0.0010		3410.0180	2511.7270	4538.3390
RESNETGAN	MLPGAN	10	−1632.6400	−2744.9600	−520.3190	−2538.2000	−725.1280	0.9932		−1900.1700	−2820.9900	−477.2790
RESNETGAN	SAGAN	10	−1526.7800	−2617.5000	−436.0570	−2473.5200	−724.6250	0.9990		−974.2620	−2589.3700	−601.7930
SAGAN	CDGSPAN	10	**10102.1800**	**9204.7460**	**10999.6100**	**9422.5590**	**10877.8800**	**0.0010**		**9743.0550**	**9119.4530**	**11180.5300**
SAGAN	CNNGAN	10	2604.3450	1274.1150	3934.5740	1525.3250	3736.5060	0.0020		2329.7140	1289.1760	3962.2360
SAGAN	DCGAN	10	7073.1760	5916.9490	8229.4030	6159.8310	8042.4900	0.0010		6976.3090	5560.9490	8328.9250
SAGAN	GraphGAN	10	5022.3060	3859.5000	6185.1120	4069.3350	5982.4190	0.0010		5170.6420	3536.2160	6241.8430
SAGAN	MLPGAN	10	−105.8640	−898.3640	686.6353	−733.4750	552.4318	0.5771		23.1387	−1191.0600	662.2354
SAGAN	RESNETGAN	10	1526.7780	436.0573	2617.4990	722.1220	2485.2190	0.0020		974.2617	601.7930	2589.3690

The nonparametric results reinforce this conclusion: the Wilcoxon one-sided p-values for the CDGSPAN comparisons are extremely small (≈ 0.001 or smaller when the alternative is stated in the direction of CDGSPAN’s advantage), and the HL median differences are likewise far below zero with bootstrap CIs that do not cross 0, confirming the effect on the median scale and reducing sensitivity to non-normality and outliers. Several baseline-to-baseline comparisons show CIs that straddle 0 with non-significant Wilcoxon p-values, indicating no reliable difference between those methods on the paired runs and further highlighting the separation achieved by CDGSPAN. In sum, across all paired comparisons, smaller tscore reliably corresponds to higher sample quality—i.e., generated 3D skeletons that are closer to real data—and CDGSPAN consistently achieves the lowest (best) *t*_score_ among all methods. The agreement of the mean differences, both types of 95% confidence intervals, the Wilcoxon tests, and the HL median estimates provides convergent statistical evidence that CDGSPAN generates the highest-quality samples under this metric.

The Table 8 provides a comprehensive analysis of biomechanical metrics, evaluating various models in comparison to the ground truth. The key metrics analyzed include bone length consistency, angular velocity, angular acceleration, and joint angles, alongside their deviations from ground truth data.

**Table 8:** Comparison of key bomechanical metrics between generated models and ground truth.

Model name	Average bone length consistency (mm)	Angular velocity mean squared error (MSE)	Average angular acceleration(rad/s^2^)	△Average angular acceleration(rad/ s^2^)	Average joint angles(rad)	△Average joint angles (rad)
**Ground truth**	0.0000	0.0000	−0.0008	0.0000	2.2070	0.0000
**CDGSPAN**	**31.0564**	**0.0800**	**0.0005**	**0.0013**	**2.1657**	**0.0413**
**MLPGAN**	245.2761	0.9421	0.0075	0.0083	1.1189	1.0881
**DCGAN**	107.8953	0.6637	0.0011	0.0019	1.8883	0.3187
**CNNGAN**	167.7284	0.9952	−0.0045	0.0038	1.5367	0.6703
**RESNETGAN**	177.6498	1.0322	0.0020	0.0028	1.3200	0.8871
**SAGAN**	152.7105	0.9892	−0.0054	0.0046	1.0666	1.1404
**GraphGAN**	135.1062	1.3604	0.0061	0.0069	2.0495	0.1575

Average bone length consistency (mm): The consistency of bone lengths across different models shows substantial variation. CDGSPAN, with an average of 31.0564 mm, exhibits a deviation compared to the ground truth (0.0000 mm), indicating a certain mismatch in the generated bone lengths. In contrast, models like MLPGAN and DCGAN show much higher discrepancies (245.2761 mm and 107.8953 mm, respectively), suggesting poor biomechanical alignment in terms of bone structure. This could affect the physical plausibility of generated poses.

Angular velocity mean squared error (MSE): The angular velocity MSE quantifies the accuracy of the generated models in replicating the ground truth data’s dynamic motion. CDGSPAN achieves the best performance with an MSE of 0.0800, closely aligning with the real angular velocities. On the other hand, models such as MLPGAN (0.9421) and GraphGAN (1.3604) demonstrate significantly larger discrepancies. These higher values indicate that these models fail to match the dynamic behavior of the ground truth, which directly impacts their dynamic plausibility and smoothness, leading to less realistic motion trajectories.

Average angular acceleration (rad/s²): Angular acceleration measures the rate of change in angular velocity over time. CDGSPAN again performs best, with a value of 0.0005 rad/s², closely matching the ground truth. In contrast, GraphGAN (0.0061 rad/s²) shows a more significant difference, suggesting less accurate replication of motion dynamics. The smaller difference observed in CDGSPAN indicates better dynamic plausibility, as it generates smoother transitions in motion, avoiding abrupt changes that would be unnatural in real-world movement.

Average angular acceleration (rad/s²): This metric measures the deviation in angular acceleration. CDGSPAN maintains a low value (0.0013 rad/s²), showing minimal fluctuation compared to the ground truth data. Models such as GraphGAN (0.0069 rad/s²) show larger discrepancies, indicating more erratic motion behavior and poor dynamic continuity.

Average joint angles (rad): Joint angles reflect the relative positions of body parts, and CDGSPAN achieves an average angle of 2.1657 rad, which is quite close to the ground truth (2.2070 rad). In comparison, GraphGAN’s joint angles are further off, with an average of 2.0495 rad. This discrepancy suggests that CDGSPAN more accurately captures the spatial arrangement of body parts, contributing to better realism in the generated poses.

Average joint angles (rad): CDGSPAN shows a minimal difference of 0.0413 rad, which is consistent with the ground truth data. Other models like GraphGAN exhibit larger differences, particularly 0.1575 rad, which again shows poorer alignment with the expected human pose, diminishing the realism and smoothness of the generated motion.

In summary, CDGSPAN emerges as the top performer in terms of biomechanical accuracy, especially when considering both bone length consistency and dynamic plausibility. It exhibits the smallest deviations in angular velocity, angular acceleration, and joint angles, thereby generating smoother, more biologically plausible motion sequences. On the other hand, models like GraphGAN and MLPGAN show larger deviations across multiple metrics, indicating that while these models may generate dynamic motion, the results may lack the realism and consistency required for biomechanical simulations, especially in areas such as joint angles and angular velocity. These discrepancies highlight the challenges in generating physically accurate and dynamically plausible motion with certain models.

## 6. Conclusion

In this article, a Constrained Dynamic Graph Spatial Perception Adversarial Network (CDGSPAN) is proposed. The model is designed to learn real 3D skeletal motion sequences and generate new sample sequences. Experimental results indicate that the generated 3D skeletal sequences closely resemble the ground-truth samples. CDGSPAN integrates dynamic graph operation and incorporates spatial constraint regularization during training, enabling the model to capture human motion features and joint-level spatial relationships from sparse real-world 3D skeletal data. Throughout the adversarial training process, the generator and discriminator are updated based on the spatial positions and angular dependencies among joints, which allows the model to learn structured kinematic patterns. After training, the model is capable of generating 3D skeletal samples from random input tensors that conform to human motion rules. Despite its effectiveness in generating valid sparse 3D skeletons, CDGSPAN still exhibits certain limitations. While CDGSPAN can yield style-consistent outputs after generation via the style-specific sample selection procedure introduced in Section 4.5, the model itself does not yet natively control style or action type at inference time. As a result, the diversity and granularity of generated poses remain constrained—particularly for specified or composite actions (e.g., bending, hand-raising, squatting, or transitions among them). This limitation stems from the absence of explicit style/action control variables in training, which prevents the generator from learning to conditionally regulate pose variation. To enable model-intrinsic control and direct style-aware outputs, more fine-grained modeling is required—e.g., introducing a conditional adversarial framework with explicit conditioning signals (style/action codes), disentangled representations, or auxiliary control heads.

CDGSPAN’s lack of native style/action control at inference has several adverse implications for deployment: target styles or composite actions cannot be specified deterministically, so outputs remain uncertain; the diversity and granularity of generated poses are limited—coverage of rare or composite motions (e.g., squat→stand with arm raise) is weak and fine details are under-expressed; reliance on post-generation style filtering introduces latency and a non-trivial rejection rate; outputs are biased toward frequent training styles, they yield poorer consistency on long-tail styles; stylistic attributes may drift across runs, reducing reproducibility; and these issues collectively constrain usability in real-time or style-critical settings such as clinical workflows and animation pipelines. If the proposed model is to generate temporally related samples, it still needs real temporal samples for guidance, which can be inconvenient.

To address this issue, future research may incorporate a broader range of 3D skeleton samples with diverse pose types for training. In addition, conditional generative mechanisms or latent space modeling techniques can be introduced to enhance controllability over motion styles and improve the diversity of generated results. The model may also suffer from overfitting or unstable training, especially when generating high-quality and biomechanically valid pose sequences. Therefore, further improvements in model architecture and integration with other generative paradigms are encouraged to enhance the realism and variety of generated samples. Future work will increasingly focus on controlling the style, type, and diversity of generated poses, thereby improving the flexibility and applicability of the model in practical scenarios.

## Supporting information

S1 Dataset The dataset file of CDGSPAN training and visualization. This dataset is described in Table 2. (PKL)

S2 File The code script for reading dataset written in Python. The code needs to run in a certain environment package.
(PY)

S3 Text The name document of the environment package required to read the dataset. This document can be quickly installed with typing “pip install - r requirements.txt” command to install the packages inside. All the files can be seen in the website: https://www.kaggle.com/datasets/luther1212/cdgspan-dataset#.
(TXT)
